# A deformation energy model reveals sequence-dependent property of nucleosome positioning

**DOI:** 10.1007/s00412-020-00750-9

**Published:** 2021-01-16

**Authors:** Guoqing Liu, Hongyu Zhao, Hu Meng, Yongqiang Xing, Lu Cai

**Affiliations:** 1 School of Life Science and Technology, Inner Mongolia University of Science and Technology, Baotou, 014010 China; 2 Inner Mongolia Key Lab of Functional Genome Bioinformatics, Inner Mongolia University of Science and Technology, Baotou, 014010 China

**Keywords:** Deformation energy, Nucleosome occupancy, Nucleosome free energy, Rotational positioning, Chromatin remodeler

## Abstract

We present a deformation energy model for predicting nucleosome positioning, in which a position-dependent structural parameter set derived from crystal structures of nucleosomes was used to calculate the DNA deformation energy. The model is successful in predicting nucleosome occupancy genome-wide in budding yeast, nucleosome free energy, and rotational positioning of nucleosomes. Our model also indicates that the genomic regions underlying the MNase-sensitive nucleosomes in budding yeast have high deformation energy and, consequently, low nucleosome-forming ability, while the MNase-sensitive non-histone particles are characterized by much lower DNA deformation energy and high nucleosome preference. In addition, we also revealed that remodelers, SNF2 and RSC8, are likely to act in chromatin remodeling by binding to broad nucleosome-depleted regions that are intrinsically favorable for nucleosome positioning. Our data support the important role of position-dependent physical properties of DNA in nucleosome positioning.

**Supplementary Information:**

The online version contains supplementary material available at 10.1007/s00412-020-00750-9.

## Introduction

Nucleosome is the first level structure of chromatin in eukaryotes, which is further packaged into a high-order chromatin structure under the control of complex mechanisms in which diverse proteins and epigenetic signals are involved (Richmond and Davey 2003; Struhl and Segal 2013; Valouev et al. 2011; Maeshima et al. 2014). Different levels of chromatin structure work cooperatively and play crucial roles in diverse functions of the chromatin such as DNA replication, DNA recombination, and gene transcription (MacAlpine and Almouzni 2013; Segal et al. 2006; Yamada and Ohta 2013). It is of great significance to explore how the chromatin is organized at the nucleosome level and how the nucleosome structure affects the expression of genetic information. Moreover, cell type–specific nucleosome organization, the dynamics of the chromatin structure in the cell division and differentiation, and disease-related aberrations in the chromatin structure are also poorly understood.

There has been a great progress in decoding the nucleosome positioning mechanism (Struhl and Segal 2013). It is widely accepted that nucleosome positioning depends on DNA sequence (Kaplan et al. 2009; Segal et al. 2006; Struhl and Segal 2013). Accordingly, a great number of sequence-dependent models were developed to predict nucleosome positioning (Anselmi et al. 1999; Bishop 2008; Chen et al. 2012; Cui et al. 2014; De Santis et al. 2010; Deniz et al. 2011; Gabdank et al. 2010; Guo et al. 2014; Kaplan et al. 2009; Liu et al. 2016; Miele et al. 2008; Morozov et al. 2009; Peckham et al. 2007; Segal et al. 2006; Sereda and Bishop 2010; Tolstorukov et al. 2007; van der Heijden et al. 2012; Xing et al. 2011; Yuan and Liu 2008; Zhao et al. 2010). Among the models, biophysical models based on DNA physical properties are not only physically interpretable but also practically useful in elucidating nucleosome positioning patterns (Anselmi et al. 1999; Bishop 2008; De Santis et al. 2010; Deniz et al. 2011; Liu et al. 2016; Miele et al. 2008; Morozov et al. 2009; Sereda and Bishop 2010; Tolstorukov et al. 2007). The DNA structure can be described as different geometric models, such as worm-like chain and elastic rod (Dickerson 1989; Olson et al. 1998; Olson et al. 2001; Peters and Maher 2010). Among the elastic rod models, base-pair step models achieved a great success in modeling nucleosome structure and organization. For example, some are able to estimate nucleosome stability and nucleosome free energy (Anselmi et al. 1999; Sereda and Bishop 2010), while some others can predict nucleosome rotational positioning (Liu et al. 2016).

Although the biophysical models based on DNA physical properties presented previously achieved a great success in predicting nucleosome positioning, the role of physical properties of DNA in determining nucleosome positions still remains unclear in several aspects. For example, what sequence-dependent features are associated with MNase-sensitive nucleosomes? Possible effects of DNA sequence on NDR formation and nucleosome remodeling need to be explored from the perspective of energetics; DNA physical property–based models may be more robust in predicting nucleosome positions than machine learning methods, the accuracy of which can be affected by the noise in the training data introduced by MNase cleavage bias. In this report, we present a deformation energy–based model for predicting nucleosome positioning, in which a position-dependent structural template for the nucleosomal DNA is used to estimate the deformation energy for DNA sequence. The results indicate that our model is successful in predicting nucleosome occupancy, nucleosome rotational positioning, and nucleosome formation free energies. We also reported an interesting link between intrinsic sequence preference and nucleosome sliding around nucleosome-depleted regions (NDRs).

## Materials and methods

### Materials

Data used in this study include genome-wide normalized nucleosome occupancy (in vitro and in vivo) in *Saccharomyces cerevisiae* (Kaplan et al. 2009), nucleosome dyad scores and occupancy from H3Q85C cleavage map (Chereji et al. 2018), nucleosome positions from H4S47C unique map (Brogaard et al. 2012), genomic coordinates for NDRs and flanking − 1/+ 1 nucleosomes (Chereji et al. 2018), transcription start sites (TSS) defined in Liu et al. (2018), DNA sequences used to assemble 20 nucleosomes in vitro (Cui et al. 2014), sequences used in nucleosome re-constitution in vitro and corresponding free energy data (Shrader and Crothers 1989; Thåström et al. 1999), genome-wide ChIP-seq data for remodelers (Parnell et al. 2015), MNase-sensitive particle data (Chereji et al. 2017; Kubik et al. 2015), absolute occupancy data (Oberbeckmann et al. 2019), and high-resolution chemical map for *Schizosaccharomyces pombe* (*S. pombe*) (Moyle-Heyrman et al. 2013). The complete genome of *Saccharomyces cerevisiae* was obtained from UCSC (http://genome.ucsc.edu/).

### Deformation energy model

**Fig. 1:**
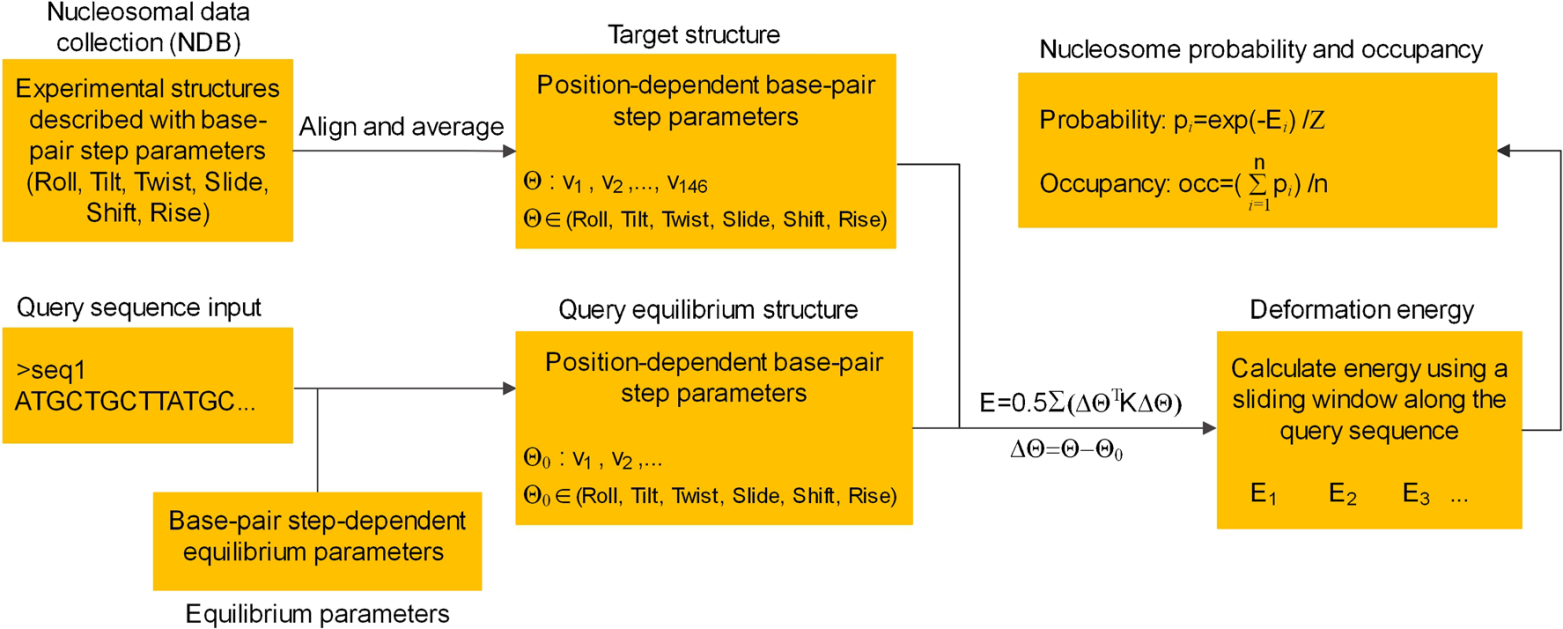
The flowchart of the position-dependent deformation energy model for predicting nucleosome occupancy

Our model begins with a deformation energy calculation for a query sequence of 147 bp. The key steps of deformation energy calculation include structural template representation for the nucleosomal DNA, equilibrium structure representation for the query sequence, and the calculation of the deformation energy for the query sequence using a harmonic model. The flowchart of the model is shown in Fig. 1, and the detailed information of the model is listed below.

Step 1. template structure representation for the nucleosomal DNA

Given the experimentally observed periodical distribution of the base-pair parameters, particularly the roll, tilt, and slide, along the nucleosomal DNA (Richmond and Davey 2003; Vasudevan et al. 2010), we adopt an assumption that the structure of the nucleosomal DNA (template or target structure) is position-dependent. The position here represents the order of the base-pair steps (or dinucleotide steps) along the nucleosomal DNA. This positional dependency, in fact, captures the relationship between the phase of the base-pair steps relative to the nucleosome dyad and the bending property of DNA, which facilitates the tight wrapping of DNA around histones for nucleosome formation (Richmond and Davey 2003).

An important issue worthy of noting in this step is that the target structure is assumed, due to the paucity of nucleosome crystal structures, to be independent of the type of dinucleotides in the nucleosomal DNA. By this we mean that if a sequence of 147 bp is presumed to form a nucleosome, it needs to adopt the unique target structure after wrapped around a histone octamer, regardless of the type of the dinucleotide at a particular position. The unique target structure (Table S1) represented by position-dependent base-pair step parameters is obtained by averaging the experimental structures of 6 nucleosomes in PDB (PDB accession codes were listed below Table S1) (Berman et al. 2000). In the averaging process (Fig S1), in order to obtain a symmetric template, we averaged the two halves of the structure relative to the dyad position. Note that the shift and tilt parameters in nucleosomes show anti-symmetrical relationship relative to dyad position, and therefore, their signs for half of the structure need to be changed manually in the averaging process. After averaging the two halves, obtained rotational parameters (tilt, roll, and twist) is smoothed by using a cubic spline fit to reduce the noise in their 10-bp periodical oscillation. Translational parameters (shift, slide, and rise) do not show strong oscillation pattern and therefore are not smoothed. The final template structure obtained in this way ensures that DNA deformation energy calculated for either Watson or Crick strand of a DNA segment is the same. The six structures are selected using the following criteria: length of nucleosomal DNA is 147 bp; resolution is higher than 3 Å; no RNA molecules in the structure; no non-histone proteins in the structure (like remodeler and transcription factors); no cancer-related mutations in the histones; no tissue-specific histone variants; no adducts of ruthenium (II)-toluene PTA complex in the structure. These criteria are used to capture more reliable properties of canonical nucleosomes.

Step 2. equilibrium structure representation for a query sequence

For a query sequence, 147 bp in length, its equilibrium structure is represented by the equilibrium dinucleotide–dependent structural parameters, which were taken from our previous study (Liu et al. 2019) (Table S2). These parameters were estimated by averaging the values of the base-pair step parameters derived from the protein-DNA crystal structures in the NDB database (http://ndbserver.rutgers.edu/).

Step 3. deformation energy calculation for the query sequence

As previously done (Deniz et al. 2011; Morozov et al. 2009), we calculated the deformation energy of a query sequence using a harmonic model (Eq. (1)). The deformation energy here means the elastic energy of the query sequence when it is, presumably, deformed by the DNA-histone interactions from equilibrium state to the nucleosomal target structure. The force constants for all the base-pair steps were also taken from our previous study (Liu et al. 2019).

1 $$ E=\frac{1}{2}\sum {\Delta \Theta}^T\mathrm{K}\Delta \Theta $$

In this equation, ΔΘ = Θ − Θ_0_ is column vector composed of the deviations of six base-pair step parameters (Θ = roll, tilt, twist, slide, shift, and rise) from their equilibrium values (Θ_0_) at a base-pair step position in the query sequence; Supposing the query sequence fragment forms a nucleosome, Θ denotes position-dependent base-pair step parameters in the nucleosomal target structure; *T* means transpose of the matrix; K is a 6 × 6 symmetric matrix composed of force constants for each dinucleotide (Table S3), in which the diagonal elements are for 6 base-pair step parameters and the others for inter-parameter couplings; because the relatively straight ends of nucleosome DNA contribute little to DNA deformation, we used a 129-bp window (*m* = 129) in deformation energy calculation. In this case, the nucleosomal target structure corresponds to the central *m*-bp region of the original 147-bp target structure (see step 1). In some cases (e.g., Fig. 3), a window of 101 bp is used for calculating rotational deformation energies, just because it is at least 10 bp smaller than the shortest sequences analyzed in this study and able to give a prediction to rotational setting of nucleosomes in at least one helical turn.

Step 4. nucleosome occupancy estimation

Once the DNA deformation energy is obtained, the probability of a nucleosome dyad being at a position along the underlying DNA can be estimated by using Boltzmann distribution law.

2 $$ p=\frac{e^{-\beta E}}{Z} $$

where partition function *Z* =  ∑ *e*^−*βE*^. The summation is over the whole genome. For computational simplicity, we assume *β* = 1.

Nucleosome occupancy at a genomic position is then defined as the mean of the dyad probabilities in an *n*-bp window spanning the position.

3 $$ occ=\left({\sum}_{i=1}^n{p}_i\right)/n $$

Due to averaging effect, a larger window size (e.g., *n* = 147 bp) in nucleosome occupancy calculation cannot predict the narrow-scaled nucleosome depletion at linker regions well. On the other hand, much smaller window (< 20 bp) would introduce larger noise in predicted occupancy. And thus we used *n* = 51 nucleosome occupancy calculation, which is a tradeoff between the capacity of detecting narrow NDRs and reducing noise.

### Grand canonical model for simulating regular positioning of nucleosomes around NDRs

Besides the simple Boltzmann model above, a grand canonical model as described in literatures (Liu et al. 2016; Morozov et al. 2009) but with a predefined energy barrier at NDRs was used to simulate nucleosome spacing pattern in vivo. In the model, steric exclusion between adjacent nucleosomes, energy barrier, and chemical potential was considered. The energy barrier in the model was designed similarly as in the literature (Chereji et al. 2018), which consists of flat energy at the center of NDRs and flanking two half Gaussian-shape boundaries. Detailed information about parameter training and energy barrier in the model were described in Supplementary Methods (Supplementary Information).

## Results and discussion

The deformation energy model in this study is designed to estimate elastic energy for DNA segment by comparing equilibrium structure for the DNA and a predefined position-dependent nucleosomal template structure, which is obtained by averaging over high-resolution crystal structures of nucleosomes. Base-pair step description of DNA structure is used, and the deformation energy refers to the elastic energy in DNA aligned to the template structure. A simple physical principle that underlies the deformation energy model is that DNA deformation is sequence-dependent and low deformation energy indicates a low energy cost in nucleosome formation and, in other words, a high preference for nucleosome forming. The validation and application of the model are provided below.

### Rotational deformation energy is predictive of nucleosome rotational positioning

**Fig. 2:**
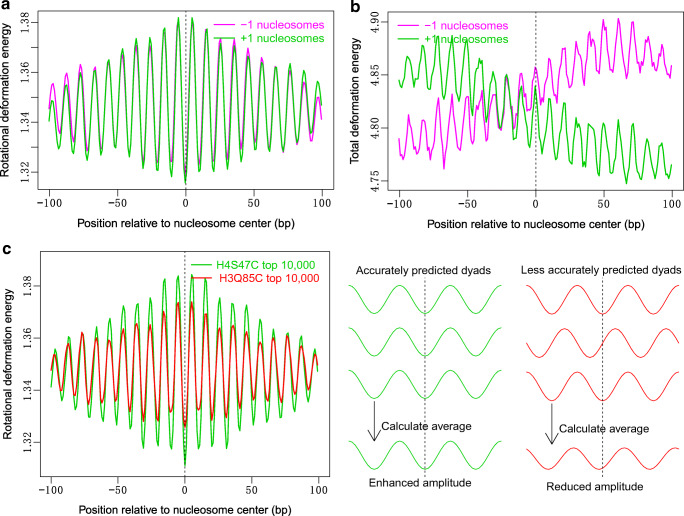
Local minimum of rotational deformation energy predicts rotational positioning. **a** Distribution of rotational deformation energy that involves three rotational base-pair step parameters (roll, tilt, and twist) at − 1/+ 1 nucleosomes. **b** Total deformation energy. **c** Comparison of rotational deformation energy between H3Q85C map (Chereji et al. 2018) and H4S47C map [Brogaard et al. 2012). As illustrated in the right panel (**c**), smaller oscillation amplitude in rotational deformation energy for H3Q85C map may indicate a reduced power in capturing precise dyad positions

It was reported that nucleosomes surrounding the gene upstream NDRs have a strong periodical oscillation in their bending energy profile, which act as a good predictor of rotational positioning (Liu et al. 2018). Rotational positioning of nucleosomes at gene start is important, as it is closely related to nucleosome sliding (Chereji et al. 2018; Liu et al. 2018), which may frequently happen during gene transcription. To test if our model could capture the rotational positioning pattern at gene start, we analyzed deformation energy profiles for + 1/− 1 nucleosomes whose genomic positions were identified with a base-pair resolution (Chereji et al. 2018). The results show that, when aligned at their centers respectively, the well-positioned nucleosomes (+ 1/− 1 nucleosomes) have a strong ~ 10-bp periodicity and large oscillation amplitude in the rotational parameter-associated deformation energy, which is the energy component corresponding to three rotational angles (roll, tilt, and twist). The + 1/− 1 nucleosomes also display local energy minima at their central positions (Fig. 2a). The positions with local minima of rotational deformation energy, differing by the multiples of the DNA helical repeat (~ 10 bp), indicate a high probability that a nucleosome’s center occurs at that positions with the same rotational setting, where the major groove of the DNA faces the histones. In contrast, when analyzed total deformation energy, no local energy minimum is observed at the centers of + 1/− 1 nucleosomes, but instead a local peak in oscillated deformation energies appears at the centers (Fig. 2b). In addition, the overall increasing trend of the deformation energy from upstream to downstream direction (Fig. 2b) suggests the decreasing capacity of nucleosome forming along this direction, which is caused by the inhibitory effect of NDRs downstream the − 1 nucleosomes. Similarly, the higher deformation energies upstream the + 1 nucleosomes is also attributable to NDRs.

We have also predicted with less than 2-bp error the rotational positions of 17 out of 20 nucleosomes assembled in vitro by using rotational deformation energy (Fig S2). This prediction accuracy is comparable to that of previously developed models (15–16 successful predictions) (Alharbi et al. 2014; Cui et al. 2014). Furthermore, our model achieved success on 4 of the 5 nucleosome positions (2 positions on pGUB, 1 on fragment-67, and 1 on chicken β-globin) that were inaccurately predicted in Cui et al. (2014), indicating that our model acts at least as a complementary method. Similarly, rotational deformation energy is able to predict the rotational positioning of nucleosomes collected from PDB (only 11 nucleosomes retained in this analysis after excluding nucleosomes with 100% DNA sequence identity), with only one exception for 3REJ (Fig S3). Furthermore, prediction is successful not only for 147-bp nucleosomes but also for 145-bp and 146-bp nucleosomes (Fig S3). Taken together, the results presented here by our position-dependent model support that the rotational deformation energy, which is related to DNA bending property, is able to capture the rotational positioning of nucleosomes.

The performance of the model in identifying rotational positions of nucleosomes allows one to get a quick check about the reliability of the results of the experiment designed to capture dyad position (or rotational positioning) of nucleosomes in vivo. We applied the model to H3Q85C map (Chereji et al. 2018) and H4S47C map (Brogaard et al. 2012) and compared rotational deformation energy between the two maps. Both H3Q85C and H4S47C maps were generated by chemical cleavage methods, which were designed to precisely map nucleosome positions with base-pair resolution. In H4S47C chemical mapping method, site-specific DNA cleavage near nucleosome dyads is achieved by hydroxyl radicals generated by Fenton reactions, which occur at the desired locations due to substitution of wild-type histone H4 gene with a H4S47C mutant in the presence of copper ion and peroxide. The ends of the DNA cleavage fragments estimate well the nucleosome dyad positions. By using the same strategy, H3Q85C mapping method, in which wild-type histone H3 gene is substituted with a H3Q85C mutant, can generate a 51-bp DNA cleavage fragment from single nucleosome, which is long enough to be sequenced and uniquely mapped for precise nucleosome mapping. In the H4S47C unique nucleosome map (Brogaard et al. 2012), the authors reported a set of selected strongest nucleosome positions while in the H3Q85C map (Chereji et al. 2018), averages of nucleosome clusters were reported. To make our comparison more rational, we first called a set of unique nucleosomes similarly as in H4S47C chemical mapping (Brogaard et al. 2012) from the H3Q85C map (Chereji et al. 2018) by using the same unique nucleosome calling approach (Xi et al. 2014), where the dyad scores from H3Q85C map were used as input. We obtained a unique nucleosome map composed of 72,438 nucleosomes (Table S10), in which adjacent nucleosomes are allowed to overlap at most 40 bp. Then, we found that the average profile of the rotational deformation energy for top 10,000 nucleosomes derived from the unique H3Q85C map has smaller oscillation amplitude than that of H4S47C map, and the small amplitude is likely to indicate a reduced power in capturing the precise dyad positions (Fig. 2c). This is unexpected because H3Q85C map, as discussed previously (Chereji et al. 2018), is able to give more accurate inference of nucleosome positions than H4S47C map. A possible explanation for this result is although the H3Q85C-derived reads are likely to give more reliable information about nucleosome dyads than H4S47C-derived reads, the read mapping and subsequent mathematical inference of nucleosome dyad positions based on read enrichment might be responsible for the unexpected result. Of course, it cannot be ruled out that the discrepancy from expectation might also result from unknown shortcoming of our model.

### Rotational deformation energy is predictive of nucleosome free energy

**Fig. 3:**
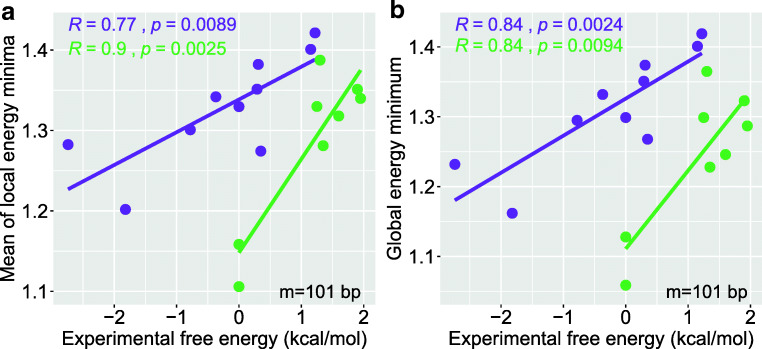
The correlation between rotational deformation energy minima and experimentally determined nucleosome free energies. Two datasets (Thåström et al. 1999; Shrader and Crothers 1989) were analyzed and represented in two colors, and the data in purple correspond to salt gradient dialysis approach (Thåström et al. 1999) for in vitro nucleosome re-constitution, while the data in green correspond to histone exchange approach (Shrader and Crothers 1989). **a** Global energy minimum is used to calculate the correlation. **b** Average of the local energy minima is used to calculate the correlation. Local energy minima are selected as the minimal values in a sliding window of 12 bp, which is a little larger than DNA helical turn, so as to capture the most probable bending orientation of DNA in a nucleosome

The bending energy minima are also predictive of the relative magnitude of nucleosome formation free energies for different DNA sequences (Liu et al. 2018). To test how our model performs in this aspect, we have calculated the rotational deformation energies for each sequence by using a 101-bp sliding window, and found a significant correlation between the average of local energy minima and experimentally determined nucleosome-forming affinity (Fig. 3a). Global minimum in rotational deformation energies also correlates strongly with the experimental free energy data (Fig. 3b). These improved predictions as compared with previous models (Liu et al. 2018; Morozov et al. 2009) also confirm the power of the present model. Note that because our model is based on crystal structures of nucleosomes, which were reconstituted by salt gradient dialysis, testing of our predictions on nucleosomes also assembled in vitro by salt gradient dialysis is less telling than testing on in vivo mapped nucleosomes.

Moreover, intrinsic cyclizability which is estimated by Loop-seq is a measure of DNA bendability (Basu et al. 2020) and closely related to DNA rotational deformation energy. After identifying the accurate center of the MNase-seq-identified nucleosomes (Kubik et al. 2015) by mapping them to H3Q85C map (Chereji et al. 2018) (see Supplementary method for details), we show that (1) fragile − 1 nucleosomes have higher rotational deformation energies (low bendability) than stable − 1 nucleosomes (Fig S5A, Wilcoxon test: *p* = 0.002 at dyad positions), which is consistent with the previous finding that fragile − 1 nucleosomes have more rigid DNA than stable − 1 nucleosomes (Basu et al. 2020). Furthermore, a stronger 10-bp oscillation in rotational deformation energy for stable nucleosomes suggests a stronger bending anisotropy (Fig S5A); (2) consistent with the detected high intrinsic cyclizability for the nucleosome group which has high NCP (Nucleosome Center Positioning) scores (Basu et al. 2020), we predicted lower values of minimal rotational deformation energy for high NCP-score group than low NCP-score group (Fig S5B, Wilcoxon test: *p* < 2.2e−16), suggesting the high DNA deformability of the high NCP-score group.

### Prediction of nucleosome occupancy

We performed genome-wide prediction of nucleosome occupancy in budding yeast and compared with both MNase-seq maps (Kaplan et al. 2009; Chereji et al. 2017) and H3Q85C map (Chereji et al. 2018). The correlations with Kaplan’s maps are significant (Table S4, *R* ≈ 0.70 in vitro; *R* = 0.52 in vivo). The results also show that the correlation with MNase-seq maps differs as the MNase concentration changes (Table S4). To be specific, significant positive correlations were observed for extensively digested nucleosome map, but no significant or even negative correlations were observed for moderately or mildly digested maps. The major difference between nucleosome maps obtained with variable concentration of MNase is MNase-sensitive particles bound to chromatin (such as MNase-sensitive nucleosomes) are largely digested in extensively digested chromatin but less digested in the case of low MNase concentration (Chereji et al. 2017). Therefore, the results suggest that although our sequence-dependent deformation energy model can predict MNase map obtained with high MNase concentration, it is unable to capture the positions of MNase-sensitive molecules on the genome. This is because MNase-sensitive nucleosomes are located preferentially in AT-rich regions (Chereji et al. 2017), which tend to be predicted as nucleosome-unfavorable regions by most of the published sequence-dependent models. Weak positive correlations were obtained between prediction and H3Q85C map (Table S4).

**Fig. 4:**
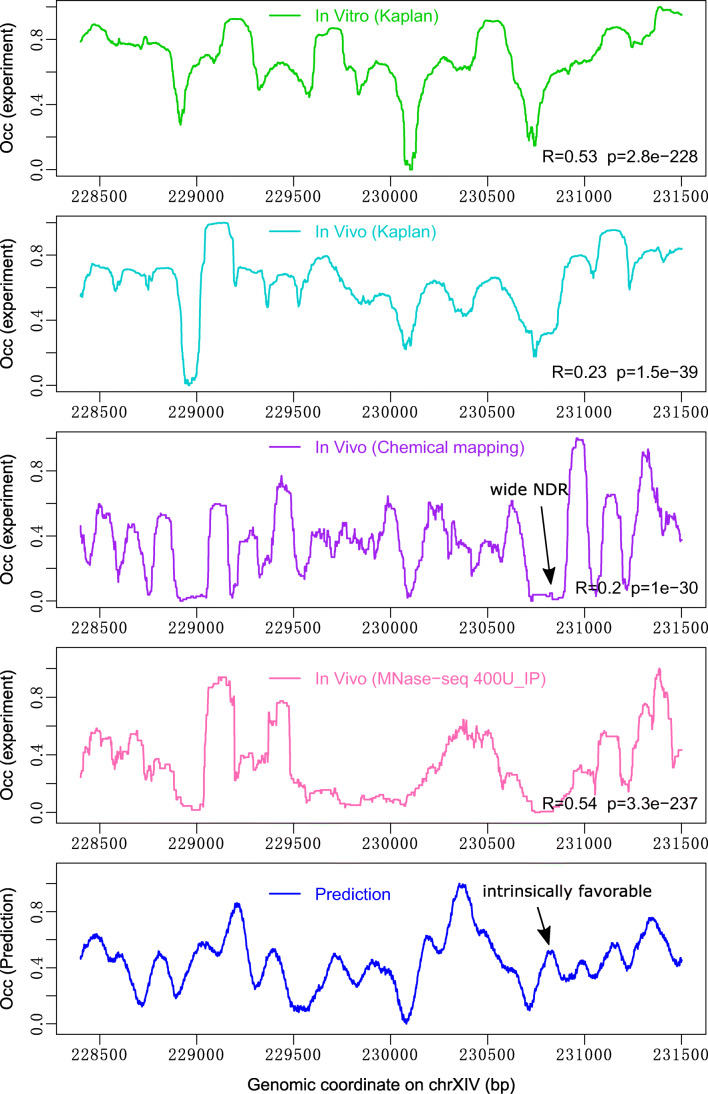
Nucleosome occupancy profile at a genomic region in budding yeast. Tracks 1 and 2: nucleosome occupancy in vitro and in vivo (Kaplan et al. 2009); track 3: nucleosome occupancy from H3Q85C map (Chereji et al. 2018); track 4: nucleosome occupancy from MNase map (Chereji et al. 2017); track5: predicted nucleosome occupancy. A wide NDR in vivo predicted to be “intrinsically favorable” for nucleosome positioning was indicated. Pearson correlations of prediction with experimental maps were shown in the corresponding tracks

An exemplary region previously reported in Chereji et al. (2018) is shown in Fig. 4 to give a visual check about the agreement between prediction and experimental data. As described above, there is a positive correlation between prediction and extensively digested nucleosome map, and the regions of MNase-sensitive nucleosomes are predicted to be nucleosome unfavorable. Besides, our sequence-based model is less powerful in predicting nucleosome occupancy in vivo, emphasizing the effect of factors other than DNA sequence preference on the nucleosome positioning in vivo. For example, we predicted an “intrinsically favorable region” for nucleosome positioning at a wide NDR in vivo (Fig. 4: track 3–5). The factors that may be responsible for this discrepancy will be discussed later in detail.

**Fig. 5:**
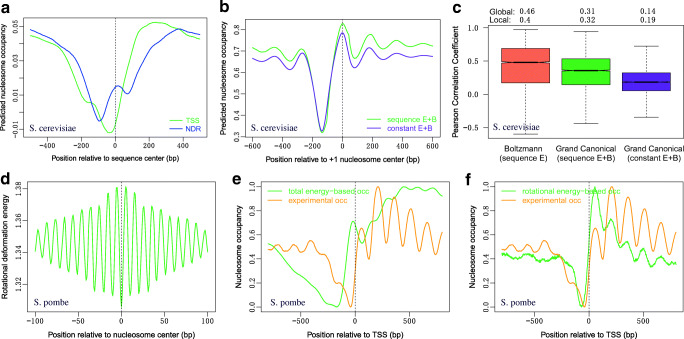
Prediction of nucleosome occupancy at transcription start sites (TSS) and nucleosome-depleted regions (NDR). **a** Boltzmann model prediction. **b** Energy barrier–based model reproduced the statistical positioning pattern of nucleosomes around NDRs. Energy barrier was imposed at NDRs located between + 1/− 1 nucleosomes. **c** Among barrier models, the one considering sequence-dependent deformation energies at non-NDR outperforms constant energy–based model but has weaker power than the simple Boltzmann model in predicting, individually, the nucleosome occupancies within 500 bp downstream regions from + 1 nucleosome dyads. Mean of the correlation coefficients for individual regions and global correlation coefficients for the regions was shown. Experimental nucleosome occupancy used in the correlation analysis was taken from MNase-seq nucleosome map (400U-IP) (Chereji et al. 2017). **d** Local minima of the rotational deformation energy coincide with the nucleosome center in *S. pombe*. **e**–**f** Total deformation energy–based nucleosome occupancy and rotational deformation energy–based nucleosome occupancy at TSS in *S. pombe*. Note that only **d**–**f** refer to *S. pombe*, and all the other results for genomic regions throughout this study correspond to *S. cerevisiae*

The present deformation energy model, as many other published models, reproduced the nucleosome depletions at the NDR upstream transcription start sites (Fig. 5a). Note that predicted nucleosome valley is not located at the center of NDRs, but instead a moderate nucleosome-forming preference is observed at the center of NDRs. This might be related to nucleosome remodeling at wide NDRs, and will be discussed lately in detail.

A simple Boltzmann model based purely on DNA deformation energy is unable to reproduce the regularly spaced nucleosome pattern around the gene start (Fig. 5a). Several studies indicated that energy barrier–based biophysical model can simulate the pattern (Chereji et al. 2018; Vaillant et al. 2010). Using a similar biophysical model in which artificial energy barrier was adopted at the NDRs located between + 1/− 1 nucleosomes (Supplementary method), we reproduced the statistical positioning pattern of nucleosomes around NDRs (Fig. 5b). Among barrier models, the one considering sequence-dependent deformation energies at non-NDR is slightly better than constant energy–based model. The results also show that although the energy barrier–based model can statistically reproduce the regular spacing pattern, they have weaker power than the simple Boltzmann model in predicting, individually, the nucleosome occupancy downstream the NDRs (Fig. 5c), supporting that sequence-dependent deformation energy is important for determining genome-wide nucleosome occupancy.

We also applied our model to the *Schizosaccharomyces pombe* genome, for which high-resolution chemical nucleosome map is available (Moyle-Heyrman et al. 2013). The results show that (1) as in *S. cerevisiae*, local minima of rotational deformation energy is also able to indicate the rotational setting of nucleosomes in *S. pombe* (Fig. 5d); (2) total deformation energy–based nucleosome occupancy prediction shows a peak at TSS, which does not coincide with experimental observation (Fig. 5e); (3) however, we found that rotational deformation energy–based nucleosome occupancy prediction can capture the nucleosome phasing downstream of the TSS (Fig. 5f). Collectively, the results suggest that although the impact of intrinsic DNA features is likely different between *S. cerevisiae* and *S. pombe*, it is clear that DNA bending property, which is largely relevant to rotational degrees of freedom (roll, tilt, and twist), plays a role in nucleosome positioning in both yeasts.

Note that, unless stated, the nucleosome occupancy in this study is an indicator of translational nucleosome positioning rather than absolute nucleosome occupancy (Oberbeckmann et al. 2019). We also analyzed the correlation between predicted nucleosome occupancy and absolute occupancy data (Oberbeckmann et al. 2019). Although we detected a positive correlation between our predicted nucleosome map and their MNase-seq nucleosome map, it is surprising that the overall correlations between our prediction and the absolute occupancy maps are very weak or insignificant (Table S9) except for the unexpected negative correlations for two vitro-reconstituted chromatin samples (GSM4193214, GSM4193216), suggesting that our deformation energy model is not suitable for predicting absolute occupancy. Possible reasons for this are (1) absolute occupancy contains not only the nucleosome positioning signal but also some other proteins’ binding signal (Oberbeckmann et al. 2019), resulting in the poor prediction of the absolute occupancy with our model, which is designed to capture only nucleosome positioning information; (2) it is also possible that the poor prediction is due to the shortcoming of our model, because although the absolute occupancy contains some information other than nucleosome occupancy, it is closely related to nucleosome occupancy (Oberbeckmann et al. 2019).

### MNase-sensitive nucleosomes have clear characteristics in their deformation energy

**Fig. 6:**
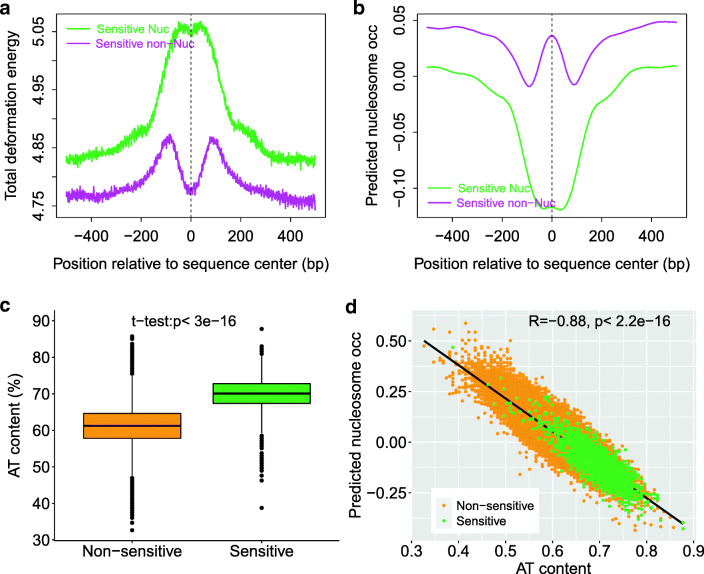
MNase-sensitive non-histone molecules have lower DNA deformation energy (**a**) and higher nucleosome forming ability than MNase-sensitive nucleosomes (**b**). AT content comparison between MNase-sensitive nucleosomes and non-sensitive nucleosomes (**c**). The correlation between predicted nucleosome occupancy and AT content (**d**). Both the correlation coefficient and regression curve in **d** are for the whole nucleosome set

MNase-sensitive nucleosomes (also called fragile nucleosomes) at stereotypical location of promoter NDRs were reported in several studies (Chereji et al. 2017, and references therein), and it was, based on the results of MNase-ChIP-seq and sonication-ChIP-seq, reported that the majority of MNase-sensitive particles at yeast promoters were non-histone protein complexes rather than nucleosomes and MNase-sensitive nucleosomes were enriched at TTS (Chereji et al. 2017). It was, however, argued that the presence of MNase-sensitive nucleosomes at gene promoters in several model organisms was well established (Kubik et al. 2017). Recently, it was revealed that fragile particles at NDRs represent the occupancy of the RSC-associated nucleosome remodeling complex and RSC-bound partially unwrapped nucleosomal intermediates (Brahma and Henikoff 2019). In order to show if the two types of MNase-sensitive particles differ in their nucleosome-forming ability, we analyzed them by using our deformation energy model. It is evident that MNase-sensitive non-histone molecules are characterized by lower DNA deformation energy and higher nucleosome-forming ability than MNase-sensitive nucleosomes (Fig. 6). In other words, our results suggest that the regions underlying the MNase-sensitive non-histone molecules located preferentially at promoter favor nucleosome forming and the regions underlying the MNase-sensitive nucleosomes located at gene ends are unfavorable for nucleosome positioning.

It is possible that the correlations regarding MNase-sensitive particles may reflect that AT-rich sequences both confer increased MNase sensitivity and unfavorable deformation energy for nucleosome formation. We tested if nucleosome’s MNase sensitivity is caused by the unfavorable deformation energy profile or by AT richness as follows. Firstly, we obtained a set of unique nucleosomes from the H3Q85C map (Chereji et al. 2018) (see supplementary method). Secondly, we mapped the previously identified MNase-sensitive nucleosomes (Chereji et al. 2017) to the unique nucleosome set, and in this way we obtained MNase-sensitive nucleosomes and non-MNase-sensitive nucleosomes from the unique nucleosome set. Note that to avoid potential bias caused by MNase-sensitive non-histone particles (Chereji et al. 2017), unique nucleosomes which have overlap with MNase-sensitive non-histone particles were excluded from analysis. Thirdly, as previously revealed by others, we showed the characteristic higher AT content of MNase-sensitive nucleosomes than non-MNase-sensitive nucleosomes (Fig. 6c). Fourthly, we showed that AT content is strongly anti-correlated with predicted nucleosome occupancy for MNase-sensitive nucleosomes (Fig. 6d), suggesting that AT content is inherently related to DNA deformation energy (and predicted nucleosome occupancy). If the MNase sensitivity of nucleosomes is dominated by unfavorable deformation energy, we could expect low predicted nucleosome occupancies for a subset of MNase-sensitive nucleosomes having relatively lower AT content. However, we did not detect such a correlation, suggesting that DNA deformation energy is unlikely to explain the MNase sensitivity of nucleosomes with lower AT content. Fifthly, we identified MNase-insensitive nucleosomes that show a similar total deformation energy profile as the MNase-sensitive nucleosomes (sampling from one standard deviation range around the mean of total deformation energy for MNase-sensitive nucleosomes), and found that they are also enriched in AT-rich sequences. Taken together, we show that not all the AT-rich nucleosomes are MNase-sensitive; not all the nucleosomes with low predicted occupancies are MNase-sensitive; AT content is inherently related to DNA deformation energy (and predicted nucleosome occupancy), and it is difficult for us to demonstrate which one of the AT content and DNA deformation dominate the MNase sensitivity.

### Chromatin remodelers may clear nucleosomes at intrinsically favorable regions

Nucleosome-depleted regions at gene promoters are of great importance to investigate chromatin remodeling activity. Two families of chromatin remodelers, ISWI and SWI, act in a combinatorial way to shape the nucleosome organization around NDRs. For example, ISWI family remodelers typically organize nucleosome arrays, while SWI/SNF family remodelers including canonical SWI/SNF remodeler and its paralog, RSC chromatin remodeling complex, typically disorganize nucleosomes and are involved in setting the size of NDR (Ganguli et al. 2014; Kubik et al. 2019; Parnell et al. 2015). The genome-wide ChIP-seq data (Ganguli et al. 2014; Kubik et al. 2019; Parnell et al. 2015) for remodeling factors provide us the possibility to investigate the sequence dependence of the chromatin remodeling activity at NDRs.

**Fig. 7:**
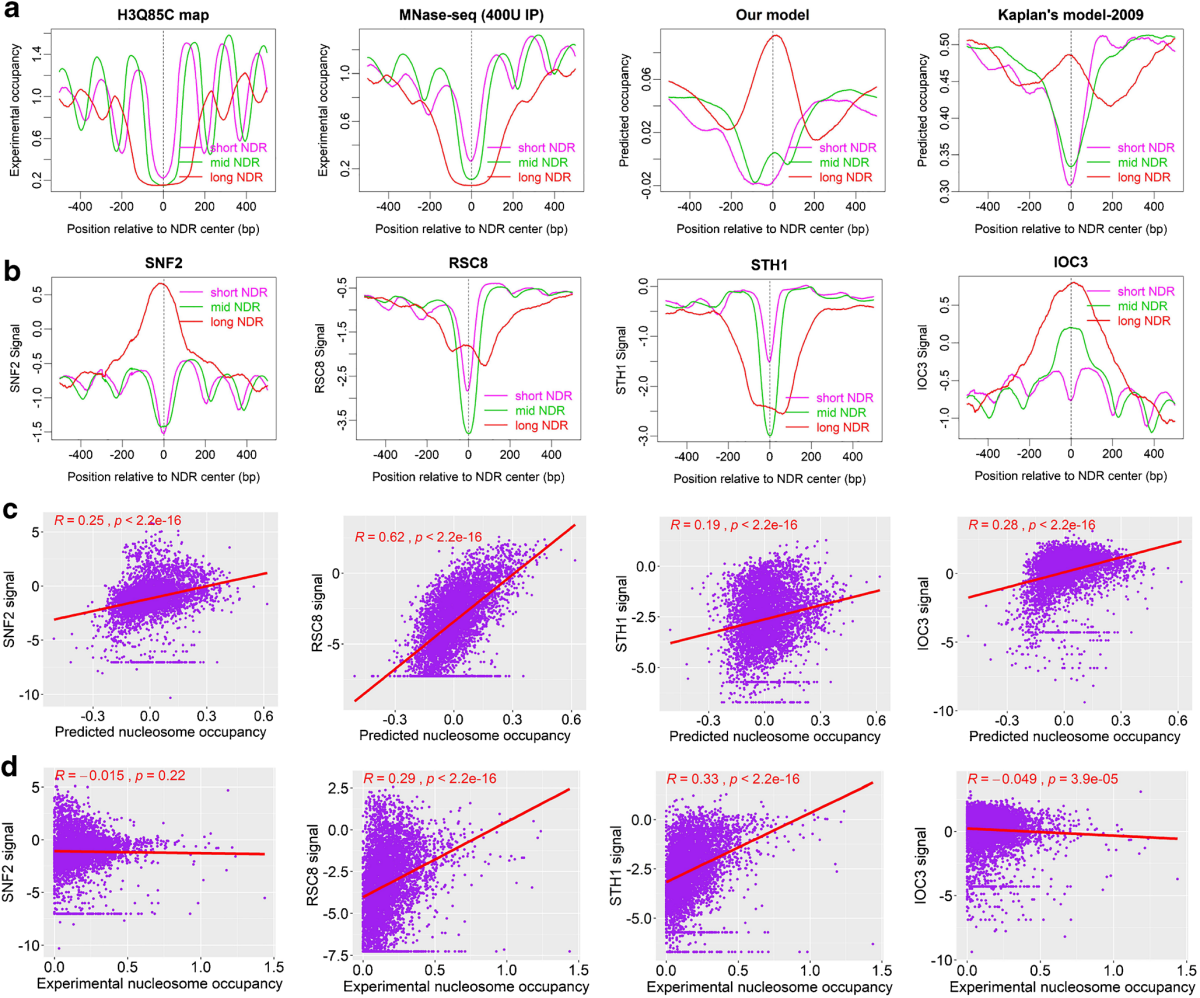
Distinct DNA intrinsic preference for nucleosome positioning at three groups of NDRs differing in size is correlated with the binding signals of remodelers. Sequence-dependent predictions (ours and Kaplan’s model) indicate that long NDRs identified in vivo tend to be intrinsically favorable for nucleosome positioning. **a** Experimental (Chereji et al. 2018; Chereji et al. 2017) and predicted nucleosome occupancy around NDRs; NDRs are classified into three groups: short NDRs ( < 100 bp, *N* = 956), mid NDRs (100–220 bp, *N* = 3710), and long NDR (> 220 bp, *N* = 876). **b** ChIP-seq signals (Ganguli et al. 2014) of four remodelers around NDRs. **c** Pearson’s correlations between predicted nucleosome occupancy and binding signals of remodelers for all the NDRs. Mean of the predicted nucleosome occupancy values in a 20-bp span centering at the NDRs is used to calculate the correlation. **d** Pearson’s correlations between experimental nucleosome occupancy and the binding signals of the remodelers as in **c**

As shown in Fig. 4, our model cannot predict wide NDRs well in vivo, suggesting that wide NDRs might be strongly regulated by epigenetic factors instead of DNA sequence preference. This raises an interesting question: how do the remodelers shape the NDRs in combination with DNA sequence preference? By analyzing the NDRs identified in vivo (Chereji et al. 2018), we discovered that, although the NDRs of different sizes are indeed deficit of nucleosomes in vivo, wide NDRs are intrinsically favorable for nucleosome positioning, while the shorter NDRs are intrinsically unfavorable (Fig. 7a). Is it possible that the “intrinsically favorable” stated here is just an inaccurate prediction of our model? To test this, we re-analyzed the NDRs by using another sequence-dependent predictive model (Kaplan et al. 2009), and the results also support that the wide NDRs are “intrinsically favorable” for nucleosome positioning (Fig. 7a). Moreover, our deformation energy model is based on DNA physical properties rather than MNase-seq data training, and it is therefore unlikely that the statement of “intrinsically favorable” is an artifact of possible sequence compositional bias, which is present in MNase-seq data due to the cleavage bias of MNase at A/T-rich sites (Chung et al. 2010; Mieczkowski et al. 2016; Xi et al. 2011).

Then, we are interested to see if the binding of chromatin remodelers is associated with the wide NDRs mentioned above. We analyzed four chromatin remodelers, SNF2, RSC8, STH1, and IOC3, the first three of which are members of SWI/SNF family and IOC3 belongs to ISWI family. Interestingly, we found distinct enrichment of SNF2, RSC8, IOC3, and STH1 at the three groups of NDRs differing in size (Fig. 7b). Specifically, SNF2 signal is weak in shorter NDRs, but preferentially bind to wide NDRs. RSC8 and IOC3 also show a higher binding signal at long NDRs than at shorter NDRs, while the binding affinity of STH1 does not differ between the three groups of NDRs. Surprisingly, the binding signals of SNF2 and RSC8 correlate more strongly with predicted nucleosome occupancy than with experimentally determined nucleosome occupancy in vivo (Fig. 7c–d), suggesting again that the co-occurrence of the remodeler signals and intrinsic preference for nucleosome positioning is unlikely to be caused by chance. Based on these results, assuming the NDRs analyzed in this study are real NDRs in vivo, we propose a biological model for chromatin remodeling: the SNF2 and RSC8 are first recruited to nucleosomes, of which the underlying DNA sequences are intrinsically favorable for nucleosome forming, assembled originally at the downstream of short NDRs probably at a particular cell cycle stage, and either move or eject the nucleosomes to produce a wider NDRs for highly transcribed genes. The remodelers would then be used as a barrier for regularly spaced nucleosome arrays surrounding the NDRs. This model is compatible with the findings that RSC8 contributes to broad NDR width (Parnell et al. 2015), and genes with broad NDRs upstream the + 1 nucleosome are highly transcribed than those with short NDRs (Chereji et al. 2018). Alternatively, although it is also theoretically possible that the SNF2 and RSC8 simply prefer binding to the wide NDRs, which subsequently act in chromatin remodeling around the NDRs, there is no reason to relate the binding of the remodelers to the intrinsic favorable property of the wide NDRs.

It worth noting that SNF2 was previously reported to clear nucleosomes from intrinsically unfavorable sites to establish NDRs at promoters (Tolkunov et al. 2011). However, this conclusion is based on the comparison of nucleosome occupancy at gene promoters between SNF2-mutant and wild-type yeast, without considering the different NDR width. Our results imply that at least a subset of nucleosomes located at intrinsically favorable sites is subject to chromatin remodelers, playing a role in the establishment of NDRs in vivo.

We have to point out that one should be cautious in interpreting the observed link between remodeler binding and DNA sequence property, because the reliability of ChIP-seq measurements of remodeler binding in vivo has been argued. For example, there are many false negative and misleading positive cases (Yen et al. 2012; Zentner et al. 2013). It needs further investigation to test whether the observation reflects real case or not.

### Testing template nucleosome structure by using diverse reference nucleosome sets

Template nucleosome structure in our deformation energy model (see step 1 in “Deformation Energy Model”) is inferred by averaging 6 nucleosome structures. This is not a sufficiently diverse set for the averaging of base-pair properties. We therefore tested our model by using three other template structures derived from more diverse nucleosome sets (Table S5, Table S6, Table S7). Three other template structures tested here are (1) template structure derived by averaging over 53 nucleosomes, which meet the criteria (see step 1 of our model) except allowing the length of nucleosomal DNA to be in the range of 145–147 bp. In the averaging process, a few unavailable values for base-pair step parameters are estimated by their symmetrical counterpart relative to nucleosome dyad; (2) to avoid potential unexpected smoothing effect which may be caused by aligning unequally sized nucleosomes (145–147 bp), we also used a template structure obtained from 19 nucleosomes of 146 bp; (3) a template structure derived from 24 nucleosomes of 145 bp. Note that internally crosslinked nucleosome core particles (5ONG, 5ONW, 5OMX) were not included in obtaining the above three templates.

Using the three template nucleosome structures, we obtained almost the same results for nucleosome center prediction as the six nucleosome-based template, and the predicted nucleosome occupancies based the three templates are all significantly correlated with experimental nucleosome map (Table S8). Note that, using the more diverse reference nucleosome sets, we did not obtain improved performance in the prediction of both nucleosome center and occupancy. Unexpected smoothing effect when averaging the structures of nucleosomes with different lengths (e.g., 145 bp, 146 bp, and 147 bp) might be a possible cause, which may weaken the periodical distribution of the base-pair step parameters like roll and tilt, thereby affecting deformation energy calculation.

Parameter space of DNA force constants in our model is not small, because there are 16 (10 unique types if complementary strand is considered) dinucleotide types and the force constant matrix per dinucleotide step contains 36 parameters. This may cause overfitting. To test this possibility, we randomly separated the most diverse nucleosome reference set consisting of 53 nucleosomes into two equal-sized sets, and each of which was referred to as either training set or test set in the subsequent 2-fold prediction. Training set was used to obtain a new template nucleosome structure, and nucleosome DNA sequences from test set were used for prediction. In each round of prediction, entirely identical nucleosome DNA sequences (100% sequence identity) derived from different nucleosome core particles were excluded from test set and only the results for 11 unique sequences were reported (Fig S4). Our results show that 10 out of 11 nucleosomes were predicted to have low rotational deformation energy near their center. Considering that our model also shows a good performance in the prediction of both nucleosome dyad positions and nucleosome occupancy for in vivo genomic sequences, which are independent of the nucleosome reference set, it is unlikely that our results were caused by overfitting.

To conclude, we presented a deformation energy model, which is able to predict nucleosome occupancy and more importantly performs well in predicting nucleosome free energy and rotational positioning. We also showed that the regions underlying the MNase-sensitive non-histone molecules favor nucleosome forming while the regions underlying the MNase-sensitive nucleosomes are unfavorable for nucleosome positioning. Moreover, we revealed that remodelers, SNF2 and RSC8, are likely to act in chromatin remodeling by binding to broad NDRs that are intrinsically favorable for nucleosome positioning. Our results highlight the dependency of nucleosome positioning on the DNA physical properties.

## Supplementary Information

ESM 1 (PDF 1221 kb)

ESM 2 (XLSX 13 kb)

ESM 3 (XLSX 1732 kb)
