# Supplementary material for: A deformation energy model reveals sequence-dependent property of nucleosome positioning
Source: Chromosoma. 2021 Jan 16;130(1):27–40. doi: 10.1007/s00412-020-00750-9 (PMC7889546; doi:10.1007/s00412-020-00750-9)
Supplement: Supplementary file 1 — (PDF 1221 kb) [file 412_2020_750_MOESM1_ESM.pdf]

Supplementary Information for "A deformation energy model reveals sequence-dependent property of nucleosome positioning"

Supplementary Information List:

Table S1. Position-dependent parameters of the template nucleosome structure obtained by averaging over six nucleosomes whose nucleosomal DNA length is 147bp

Table S2. Dinucleotide-dependent force constants and equilibrium structure parameters used in this study

Table S3. Full list of dinucleotide-dependent force constants

Table S4. Pearson correlations of predicted nucleosome occupancy with experimental nucleosome maps

Table S5. Position-dependent parameters of the template nucleosome structure obtained by averaging over 54 nucleosomes whose nucleosomal DNA length is in the range of 145-147bp

Table S6. Position-dependent parameters of the template nucleosome structure obtained by averaging over 19 nucleosomes whose nucleosomal DNA length is 146bp

Table S7. Position-dependent parameters of the template nucleosome structure obtained by averaging over 24 nucleosomes whose nucleosomal DNA length is 145bp

Table S8. Pearson correlations of nucleosome occupancy predicted based on different template nucleosome structures with experimental nucleosome map

Table S9. Correlations between prediction nucleosome occupancy and absolute occupancy maps (provided in a separate Excel file)

Table S10. Unique nucleosome map called from H3Q85C map (provided in a separate Excel file)

Figure S1. The averaging process used to obtain template nucleosome structure listed in Table S1. Six nucleosomal DNA structures from PDB (5AV6, 5AV8, 5AV9, 5AVB, 5OMX, 5ONW) are aligned at the dyad position, and then averaged for each of the six base-pair-step parameters (shift, slide, rise, tilt, roll and twist). In order to obtain a symmetric template, two halves of the previous-step structure are averaged relative to the dyad position (the signs of tilt and shift for the half of the structure need to be changed manually before averaging in this step). After averaging the two halves, obtained rotational parameters (tilt, roll and twist) is smoothed by using a cubic spline fit to reduce the noise in their 10-bp periodical

oscillation. Translational parameters (shift, slide and rise) do not show strong oscillation pattern and therefore are not smoothed. Finally, final template structure is obtained by copying and concatenating (shift and tilt also change signs in the duplicated copy). The final template structure obtained here ensures that DNA deformation energy calculated for Watson strand is the same with that of Crick strand.

Figure S2. Prediction of rotational positioning of 20 nucleosomes assembled in vitro. Small rotational deformation energy indicates a high probability of placing the dyad of a nucleosome at that position, where the major groove of DNA faces the histones. It is marked as “yes” if the discrepancy between the local minimum of rotational deformation energy (green solid ball) and the experimentally determined dyad positions (vertical dash line) is no more than 2 bp, otherwise, marked as “no”. The local energy minimum is defined as the smallest value in an 11-bp span centering at the dyad position and such a span is adequate to capture the most probable rotational position within the range of one DNA helical turn.

Figure S3. Prediction of rotational positioning of nucleosomes taken from PDB. Legends are the same as in Figure S2.

Figure S4. 2-Fold prediction of rotational positioning of nucleosomes taken from PDB. We randomly separated the most diverse nucleosome reference set consisting of 53 nucleosomes into two equal-sized sets, and each of which was referred to as either training set or test set in the subsequent 2-fold prediction. Training set was used to obtain a new reference nucleosome structure, and nucleosome DNA sequences from test set were used for prediction. In each round of prediction, entirely identical nucleosome DNA sequences (100% sequence identity) derived from different nucleosome core particles were excluded from the test set and only the results for 11 unique sequences were reported. Other legends are the same as in Figure S2.

Figure S5. The local minima in rotational deformation energy are predictive of nucleosome-forming preference. (A), rotational deformation energy for fragile -1 nucleosomes and stable -1 nucleosomes (Kubik et al. 2015); (B), rotational deformation energy for low NCP-score (Nucleosome Center Positioning Score) group and high NCP-score group (Brogaard et al. 2012).

Supplementary Methods:

1. Grand canonical model for nucleosome positioning
2. Calling unique nucleosomes from the H3Q85C map and mapping MNase-sensitive nucleosomes to the unique map

Table S1. Position-dependent parameters of the template nucleosome structure obtained by averaging over six nucleosomes whose nucleosomal DNA length is 147bp

Step position	Shift	Slide	Rise	Tilt	Roll	Twist
1	-0.669	-0.921	3.212	2.055	2.560	34.050
2	0.810	0.160	3.267	1.473	3.266	34.521
3	-0.678	0.351	3.348	-1.579	3.964	33.648
4	-0.353	0.178	3.193	-1.590	1.061	34.195
5	0.293	-0.619	3.135	0.388	-2.345	34.877
6	0.174	-0.007	3.735	0.969	-3.856	37.187
7	0.031	-0.886	3.204	0.626	-2.505	36.072
8	0.687	-0.267	3.285	1.152	0.693	34.734
9	-0.485	0.235	3.168	0.246	4.920	32.986
10	-0.439	0.114	3.257	-1.102	6.589	32.534
11	0.495	-0.700	3.328	0.627	6.731	32.164
12	0.559	-0.450	3.444	2.425	8.246	32.168
13	-0.570	0.397	3.174	2.174	6.573	33.526
14	0.332	1.466	3.501	1.168	2.177	36.476
15	-0.122	0.299	2.912	0.903	-1.395	37.001
16	0.299	2.132	3.430	0.167	-5.632	39.441
17	0.898	0.088	3.343	0.203	-3.883	37.718
18	-0.400	-0.390	3.488	0.020	-2.900	35.580
19	0.202	-0.886	3.315	-0.116	-1.746	33.365
20	-0.094	-0.717	3.236	1.458	3.564	31.702
21	-0.236	-0.681	3.060	1.469	7.460	30.006
22	-0.067	-0.088	3.357	0.863	10.799	29.978
23	0.429	-0.469	3.587	-0.257	11.868	30.605
24	-0.204	-0.228	3.150	-0.602	6.813	31.848
25	0.464	-0.011	3.088	1.498	2.241	33.423
26	-0.535	2.451	3.200	0.848	-4.349	37.904
27	0.278	0.242	3.365	-0.552	-6.369	38.473
28	0.317	-0.292	3.371	-0.672	-4.924	36.628
29	0.173	-0.302	3.356	-0.688	-1.553	34.884
30	-0.038	-0.582	3.474	-0.029	1.870	33.814
31	-0.402	-0.327	3.226	0.271	4.173	33.562
32	0.366	-0.021	3.451	0.042	7.331	32.018
33	-0.144	-0.485	3.117	0.241	7.573	31.913
34	0.089	-0.425	3.605	0.511	6.280	33.440
35	0.221	-0.664	3.199	-0.628	1.408	34.348
36	0.064	-0.336	3.527	-0.559	-3.176	36.108
37	-0.254	-0.273	3.180	-0.358	-6.509	37.546
38	-0.495	2.450	3.448	-0.356	-9.675	39.154
39	1.245	1.039	3.095	-0.984	-3.652	36.350
40	-1.394	0.710	3.336	-2.611	2.343	36.100

41	0.124	-0.081	3.373	-2.216	5.431	35.493
42	-0.039	-0.188	3.313	-0.723	6.995	32.589
43	0.486	-0.635	3.121	2.074	7.172	31.337
44	1.182	0.012	3.251	2.805	6.598	33.564
45	-1.284	0.753	3.523	0.267	3.094	36.990
46	0.997	0.759	3.443	0.867	-1.764	38.144
47	-1.380	0.571	3.265	1.012	-3.887	37.254
48	1.548	1.211	3.443	1.278	-3.568	38.259
49	-1.131	0.442	3.372	-0.940	-0.579	37.326
50	0.116	0.585	3.327	-0.126	2.976	34.941
51	0.092	-0.578	3.003	0.728	5.039	31.595
52	0.193	-0.151	3.320	1.599	9.099	29.746
53	-0.163	-0.007	3.487	1.212	14.396	27.359
54	0.178	0.411	3.221	-0.237	14.701	27.327
55	-0.604	0.300	3.205	-1.109	9.478	30.423
56	-0.341	0.197	3.255	-0.317	2.170	34.786
57	0.889	1.129	3.290	0.313	-5.998	38.085
58	-0.573	1.515	3.953	0.594	-9.137	40.444
59	1.282	0.760	3.614	1.052	-6.810	38.887
60	-0.962	0.652	3.392	-1.453	-0.291	35.634
61	-0.200	-0.597	3.352	-1.736	3.595	33.583
62	0.306	0.416	3.380	-0.147	6.245	33.258
63	-0.144	-0.486	3.183	0.064	6.792	32.062
64	-0.250	-0.015	3.377	0.043	7.447	32.093
65	0.538	0.129	3.191	1.101	6.900	33.744
66	0.425	0.820	3.265	0.662	3.753	36.749
67	-1.052	1.342	3.528	-1.052	-3.599	39.465
68	1.080	1.130	3.458	0.272	-7.038	38.898
69	-1.072	0.581	3.403	0.470	-5.687	37.584
70	0.849	1.673	3.547	0.543	-1.866	37.766
71	0.156	0.302	3.008	-1.254	3.768	34.553
72	-0.414	-0.272	3.391	-1.642	6.005	33.116
73	0.019	-0.672	3.317	-0.649	6.002	32.078
74	-0.019	-0.672	3.317	0.649	6.002	32.078
75	0.414	-0.272	3.391	1.642	6.005	33.116
76	-0.156	0.302	3.008	1.254	3.768	34.553
77	-0.849	1.673	3.547	-0.543	-1.866	37.766
78	1.072	0.581	3.403	-0.470	-5.687	37.584
79	-1.080	1.130	3.458	-0.272	-7.038	38.898
80	1.052	1.342	3.528	1.052	-3.599	39.465
81	-0.425	0.820	3.265	-0.662	3.753	36.749
82	-0.538	0.129	3.191	-1.101	6.900	33.744
83	0.250	-0.015	3.377	-0.043	7.447	32.093
84	0.144	-0.486	3.183	-0.064	6.792	32.062

85	-0.306	0.416	3.380	0.147	6.245	33.258
86	0.200	-0.597	3.352	1.736	3.595	33.583
87	0.962	0.652	3.392	1.453	-0.291	35.634
88	-1.282	0.760	3.614	-1.052	-6.810	38.887
89	0.573	1.515	3.953	-0.594	-9.137	40.444
90	-0.889	1.129	3.291	-0.313	-5.998	38.085
91	0.341	0.197	3.255	0.317	2.170	34.786
92	0.604	0.300	3.205	1.109	9.478	30.423
93	-0.178	0.411	3.221	0.237	14.701	27.327
94	0.163	-0.007	3.487	-1.212	14.396	27.359
95	-0.193	-0.151	3.320	-1.599	9.099	29.746
96	-0.092	-0.578	3.003	-0.728	5.039	31.595
97	-0.116	0.585	3.327	0.126	2.976	34.941
98	1.131	0.442	3.372	0.940	-0.579	37.326
99	-1.548	1.211	3.443	-1.278	-3.568	38.259
100	1.380	0.571	3.265	-1.012	-3.887	37.254
101	-0.997	0.759	3.443	-0.867	-1.764	38.144
102	1.284	0.753	3.523	-0.267	3.094	36.990
103	-1.182	0.012	3.251	-2.805	6.598	33.564
104	-0.486	-0.635	3.121	-2.074	7.172	31.337
105	0.039	-0.188	3.313	0.723	6.995	32.589
106	-0.124	-0.081	3.373	2.216	5.431	35.493
107	1.394	0.710	3.336	2.611	2.343	36.100
108	-1.245	1.039	3.095	0.984	-3.652	36.350
109	0.495	2.450	3.448	0.356	-9.675	39.154
110	0.254	-0.273	3.180	0.358	-6.509	37.546
111	-0.064	-0.336	3.527	0.559	-3.176	36.108
112	-0.221	-0.664	3.199	0.628	1.408	34.348
113	-0.089	-0.425	3.605	-0.511	6.280	33.440
114	0.144	-0.485	3.117	-0.241	7.573	31.913
115	-0.366	-0.021	3.451	-0.042	7.331	32.018
116	0.402	-0.327	3.226	-0.271	4.173	33.562
117	0.038	-0.583	3.474	0.029	1.870	33.814
118	-0.173	-0.302	3.356	0.688	-1.553	34.884
119	-0.316	-0.292	3.371	0.672	-4.924	36.628
120	-0.278	0.242	3.365	0.552	-6.369	38.473
121	0.535	2.451	3.200	-0.848	-4.349	37.904
122	-0.464	-0.011	3.088	-1.498	2.241	33.423
123	0.204	-0.228	3.150	0.602	6.813	31.848
124	-0.429	-0.469	3.587	0.257	11.868	30.605
125	0.067	-0.088	3.357	-0.863	10.799	29.978
126	0.236	-0.681	3.060	-1.469	7.460	30.006
127	0.094	-0.717	3.237	-1.458	3.564	31.702
128	-0.202	-0.886	3.315	0.116	-1.746	33.365

129	0.400	-0.390	3.488	-0.020	-2.900	35.580
130	-0.898	0.088	3.343	-0.203	-3.883	37.718
131	-0.299	2.132	3.430	-0.167	-5.632	39.441
132	0.122	0.299	2.912	-0.903	-1.395	37.001
133	-0.332	1.466	3.501	-1.168	2.177	36.476
134	0.571	0.397	3.174	-2.174	6.573	33.526
135	-0.559	-0.450	3.444	-2.425	8.246	32.168
136	-0.495	-0.700	3.328	-0.627	6.731	32.164
137	0.439	0.114	3.257	1.102	6.589	32.534
138	0.485	0.235	3.168	-0.246	4.920	32.986
139	-0.687	-0.267	3.285	-1.152	0.693	34.734
140	-0.031	-0.886	3.204	-0.626	-2.505	36.072
141	-0.174	-0.007	3.735	-0.969	-3.856	37.187
142	-0.293	-0.619	3.135	-0.388	-2.345	34.877
143	0.353	0.178	3.193	1.590	1.061	34.195
144	0.678	0.351	3.348	1.579	3.964	33.648
145	-0.810	0.160	3.267	-1.473	3.266	34.521
146	0.669	-0.920	3.212	-2.055	2.560	34.050

Note: the PDB codes of the nucleosomes used to obtain the template structure listed in this table are: 5AV6, 5AV8, 5AV9, 5AVB, 5OMX, 5ONW. These base-step parameters are symmetric relative to nucleosome dyad position (shift and tilt are anti-symmetric).

Table S2. Dinucleotide-dependent force constants and equilibrium structure parameters used in this study

	Force constants						Equilibrium parameters					
step	k(Shift)	k(Slide)	k(Rise)	k(Tilt)	k(Roll)	k(Twist)	Shift	Slide	Rise	Tilt	Roll	Twist
AA	6.641	6.845	26.514	0.113	0.041	0.071	-0.001	-0.176	3.253	-1.365	1.049	35.031
AT	4.65	9.583	24.819	0.098	0.054	0.07	0	-0.679	3.208	0	0.612	30.726
AG	3.393	3.501	21.303	0.086	0.044	0.056	0.151	-0.222	3.321	-1.52	3.538	32.259
AC	3.981	7.099	21.977	0.071	0.066	0.064	0.287	-0.594	3.243	0.275	2.015	31.524
TA	4.101	1.847	14.18	0.064	0.031	0.048	0	0.043	3.389	0	3.506	36.94
TT	6.641	6.845	26.514	0.113	0.041	0.071	0.001	-0.176	3.253	1.365	1.049	35.031
TG	3.143	2.018	14.642	0.057	0.035	0.052	0.162	0.482	3.366	0.182	5.607	35.404
TC	5.082	4.734	24.761	0.097	0.05	0.059	0.313	-0.037	3.299	1.58	2.473	35.743
GA	5.082	4.734	24.761	0.097	0.05	0.059	-0.313	-0.037	3.299	-1.58	2.473	35.743
GT	3.981	7.099	21.977	0.071	0.066	0.064	-0.287	-0.594	3.243	-0.275	2.015	31.524
GG	3.034	2.989	19.495	0.086	0.043	0.058	-0.079	-0.166	3.362	-0.784	4.705	33.537
GC	2.618	4.176	16.983	0.065	0.055	0.059	0	-0.192	3.267	0	1.743	34.06
CA	3.143	2.018	14.642	0.057	0.035	0.052	-0.162	0.482	3.366	-0.182	5.607	35.404
CT	3.393	3.501	21.303	0.086	0.044	0.056	-0.151	-0.222	3.321	1.52	3.538	32.259
CG	3.01	2.708	14.594	0.059	0.039	0.051	0	0.443	3.292	0	6.021	33.668
CC	3.034	2.989	19.495	0.086	0.043	0.058	0.079	-0.166	3.362	0.784	4.705	33.537

Note: the full list of force constants including the cross-talk between different dinucleotide-step parameters are shown in Table S3.

Table S3. Full list of dinucleotide-dependent force constants

	k(Shift)	k(Slide)	k(Rise)	k(Tilt)	k(Roll)	k(Twist)
AA						
	6.641	0.781	1.606	-0.166	0.029	0.088
	0.781	6.845	1.055	0.039	-0.168	-0.176
	1.606	1.055	26.514	-0.773	-0.157	-0.256
	-0.166	0.039	-0.773	0.113	0.001	0.005
	0.029	-0.168	-0.157	0.001	0.041	0.023
	0.088	-0.176	-0.256	0.005	0.023	0.071
AT						
	4.65	0	0	-0.037	0	0
	0	9.583	4.335	0	-0.077	-0.096
	0	4.335	24.819	0	0.095	-0.325
	-0.037	0	0	0.098	0	0
	0	-0.077	0.095	0	0.054	0.024
	0	-0.096	-0.325	0	0.024	0.07
AG						
	3.393	-0.383	0.674	-0.142	0.056	0.085
	-0.383	3.501	1.544	-0.017	0.004	-0.108
	0.674	1.544	21.303	-0.576	-0.138	-0.421
	-0.142	-0.017	-0.576	0.086	0.006	0.009
	0.056	0.004	-0.138	0.006	0.044	0.014
	0.085	-0.108	-0.421	0.009	0.014	0.056
AC						
	3.981	1.14	0.614	-0.069	0.008	-0.003
	1.14	7.099	3.928	-0.033	0.001	-0.144
	0.614	3.928	21.977	0.097	0.231	-0.346
	-0.069	-0.033	0.097	0.071	0.004	0.004
	0.008	0.001	0.231	0.004	0.066	0.012
	-0.003	-0.144	-0.346	0.004	0.012	0.064
TA						
	4.101	0	0	-0.104	0	0
	0	1.847	0.81	0	-0.001	-0.083
	0	0.81	14.18	0	-0.168	-0.246
	-0.104	0	0	0.064	0	0
	0	-0.001	-0.168	0	0.031	0.02
	0	-0.083	-0.246	0	0.02	0.048
TT						
	6.641	-0.781	-1.606	-0.166	-0.029	-0.088
	-0.781	6.845	1.055	-0.039	-0.168	-0.176
	-1.606	1.055	26.514	0.773	-0.157	-0.256
	-0.166	-0.039	0.773	0.113	-0.001	-0.005

	-0.029	-0.168	-0.157	-0.001	0.041	0.023
	-0.088	-0.176	-0.256	-0.005	0.023	0.071
TG						
	3.143	-0.258	-0.087	-0.121	0.006	0.046
	-0.258	2.018	0.378	-0.014	0.057	-0.094
	-0.087	0.378	14.642	-0.095	-0.233	-0.269
	-0.121	-0.014	-0.095	0.057	-0.001	0.001
	0.006	0.057	-0.233	-0.001	0.035	0.02
	0.046	-0.094	-0.269	0.001	0.02	0.052
TC						
	5.082	-1.38	-1.788	-0.211	-0.029	-0.074
	-1.38	4.734	1.469	-0.083	-0.051	-0.086
	-1.788	1.469	24.761	0.776	-0.021	-0.335
	-0.211	-0.083	0.776	0.097	0.006	0
	-0.029	-0.051	-0.021	0.006	0.05	0.019
	-0.074	-0.086	-0.335	0	0.019	0.059
GA						
	5.082	1.38	1.788	-0.211	0.029	0.074
	1.38	4.734	1.469	0.083	-0.051	-0.086
	1.788	1.469	24.761	-0.776	-0.021	-0.335
	-0.211	0.083	-0.776	0.097	-0.006	0
	0.029	-0.051	-0.021	-0.006	0.05	0.019
	0.074	-0.086	-0.335	0	0.019	0.059
GT						
	3.981	-1.14	-0.614	-0.069	-0.008	0.003
	-1.14	7.099	3.928	0.033	0.001	-0.144
	-0.614	3.928	21.977	-0.097	0.231	-0.346
	-0.069	0.033	-0.097	0.071	-0.004	-0.004
	-0.008	0.001	0.231	-0.004	0.066	0.012
	0.003	-0.144	-0.346	-0.004	0.012	0.064
GG						
	3.034	0.02	1.393	-0.24	0.075	0.083
	0.02	2.989	2.015	0.01	0.046	-0.092
	1.393	2.015	19.495	-0.663	-0.054	-0.329
	-0.24	0.01	-0.663	0.086	-0.005	-0.001
	0.075	0.046	-0.054	-0.005	0.043	0.017
	0.083	-0.092	-0.329	-0.001	0.017	0.058
GC						
	2.618	0	0	-0.136	0	0
	0	4.176	2.92	0	0.188	-0.135
	0	2.92	16.983	0	0.276	-0.376
	-0.136	0	0	0.065	0	0
	0	0.188	0.276	0	0.055	0.009
	0	-0.135	-0.376	0	0.009	0.059

CA						
	3.143	0.258	0.087	-0.121	-0.006	-0.046
	0.258	2.018	0.378	0.014	0.057	-0.094
	0.087	0.378	14.642	0.095	-0.233	-0.269
	-0.121	0.014	0.095	0.057	0.001	-0.001
	-0.006	0.057	-0.233	0.001	0.035	0.02
	-0.046	-0.094	-0.269	-0.001	0.02	0.052
CT						
	3.393	0.383	-0.674	-0.142	-0.056	-0.085
	0.383	3.501	1.544	0.017	0.004	-0.108
	-0.674	1.544	21.303	0.576	-0.138	-0.421
	-0.142	0.017	0.576	0.086	-0.006	-0.009
	-0.056	0.004	-0.138	-0.006	0.044	0.014
	-0.085	-0.108	-0.421	-0.009	0.014	0.056
CG						
	3.01	0	0	-0.168	0	0
	0	2.708	0.644	0	0.042	-0.044
	0	0.644	14.594	0	-0.169	-0.383
	-0.168	0	0	0.059	0	0
	0	0.042	-0.169	0	0.039	0.019
	0	-0.044	-0.383	0	0.019	0.051
CC						
	3.034	-0.02	-1.393	-0.24	-0.075	-0.083
	-0.02	2.989	2.015	-0.01	0.046	-0.092
	-1.393	2.015	19.495	0.663	-0.054	-0.329
	-0.24	-0.01	0.663	0.086	0.005	0.001
	-0.075	0.046	-0.054	0.005	0.043	0.017
	-0.083	-0.092	-0.329	0.001	0.017	0.058

Table S4. Pearson correlations of predicted nucleosome occupancy with experimental nucleosome maps

	Nuc map (Kaplan et al. 2009)		Nuc map (Chereji et al. 2017)						Nuc map (Chereji et al. 2018)
yeast	In Vitro	In Vivo	50U Input	100U Input	200U Input	300U Input	400U Input	400U IP	H3Q85C_rep1
chrI	**0.70**	**0.51**	**-0.23**	**-0.08**	**0.03**	**0.13**	**0.32**	**0.54**	**0.22**
chrII	**0.62**	**0.49**	**-0.23**	**-0.08**	**0.02**	**0.12**	**0.30**	**0.48**	**0.26**
chrIII	**0.72**	**0.56**	**-0.23**	**-0.04**	**0.07**	**0.21**	**0.39**	**0.59**	**0.31**
chrIV	**0.69**	**0.54**	**-0.26**	**-0.09**	**0.01**	**0.11**	**0.29**	**0.47**	**0.26**
chrV	**0.70**	**0.54**	**-0.29**	**-0.12**	0.00	**0.11**	**0.30**	**0.49**	**0.27**
chrVI	**0.71**	**0.53**	**-0.26**	**-0.07**	**0.03**	**0.14**	**0.31**	**0.49**	**0.27**
chrVII	**0.69**	**0.54**	**-0.23**	**-0.07**	**0.03**	**0.13**	**0.30**	**0.47**	**0.27**
chrVIII	**0.69**	**0.52**	**-0.21**	**-0.11**	**-0.05**	0.03	**0.22**	**0.46**	**0.22**
chrIX	**0.71**	**0.53**	**-0.25**	**-0.09**	**0.01**	**0.12**	**0.31**	**0.54**	**0.27**
chrX	**0.21**	**0.14**	**-0.25**	**-0.09**	0.00	**0.13**	**0.31**	**0.51**	**0.27**
chrXI	**0.69**	**0.53**	**-0.25**	**-0.08**	**0.03**	**0.14**	**0.32**	**0.48**	**0.27**
chrXII	**0.69**	**0.53**	**0.10**	**0.11**	**0.12**	**0.12**	**0.13**	**0.14**	**0.15**
chrXIII	**0.70**	**0.53**	**-0.27**	**-0.11**	**-0.01**	**0.10**	**0.28**	**0.47**	**0.26**
chrXIV	**0.68**	**0.52**	**-0.23**	**-0.08**	**0.02**	**0.13**	**0.30**	**0.47**	**0.26**
chrXV	**0.70**	**0.54**	**-0.27**	**-0.10**	0.00	**0.11**	**0.29**	**0.47**	**0.26**
chrXVI	**0.69**	**0.53**	**-0.28**	**-0.11**	0.00	**0.11**	**0.30**	**0.48**	**0.27**

Note: significant correlations at the level $P<0.0001$ are shown in bold.

Table S5. Position-dependent parameters of the template nucleosome structure obtained by averaging over six nucleosomes whose nucleosomal DNA length is in the range of 145-147bp

Step position	Shift	Slide	Rise	Tilt	Roll	Twist
1	NA	NA	NA	NA	NA	NA
2	0.011	-0.478	3.252	2.11259	1.872463	36.26898
3	0.04	0.189	3.344	0.398182	4.495718	35.9233
4	-0.333	0.367	3.356	-1.36231	3.860232	35.58183
5	-0.009	-0.225	3.194	-1.2041	0.725613	34.68018
6	0.103	-0.166	3.314	0.190348	-1.52692	35.01532
7	0.011	-0.414	3.409	0.909122	-2.73331	35.97841
8	0.377	-0.424	3.344	1.853686	-1.66689	36.37429
9	0.235	0.034	3.303	1.725727	2.590033	35.37039
10	-0.203	0.17	3.266	-0.78146	6.259892	33.49927
11	-0.282	-0.186	3.342	-1.78178	7.115927	32.40929
12	0.248	-0.564	3.355	0.053231	8.456578	31.50373
13	0.001	-0.178	3.385	1.108579	9.720526	32.64112
14	-0.222	0.954	3.461	1.77318	5.857298	35.85245
15	0.018	1.273	3.181	1.93086	-0.25754	38.37625
16	-0.175	1.371	3.3	0.808205	-3.7237	39.75333
17	0.717	1.495	3.371	0.533061	-5.02049	39.6163
18	0.216	0.214	3.387	0.811162	-2.42103	37.27854
19	-0.054	-0.309	3.404	0.802822	-1.85335	36.71375
20	0.022	-0.334	3.308	0.923059	0.31391	35.96139
21	-0.019	-0.463	3.381	0.55713	3.827899	35.00949
22	-0.075	-0.273	3.536	-0.18549	7.94198	34.3279
23	0.031	-0.007	3.528	-0.75747	9.781789	33.70512
24	0.063	0.119	3.502	-1.45698	6.811052	34.73537
25	0.142	0.111	3.366	0.005795	2.94471	35.63072
26	-0.18	1.135	3.185	0.994014	-3.51486	38.57783
27	-0.029	1.353	3.394	-0.05697	-8.52332	40.37219
28	0.306	0.18	3.3	-0.90614	-6.24982	37.94549
29	-0.1	-0.019	3.353	-1.09893	-2.78854	37.74678
30	0.017	-0.408	3.519	-0.26113	1.358497	36.56229
31	-0.201	-0.382	3.344	-0.43049	4.633998	34.20234
32	0.023	-0.079	3.352	0.018125	7.966994	32.26486
33	0.068	-0.184	3.34	0.050273	8.675291	31.52951
34	-0.032	-0.419	3.357	0.090593	5.583793	33.22854
35	0.158	-0.466	3.393	-0.01049	1.627476	35.27851
36	0.056	-0.395	3.35	0.219715	-1.6971	36.04724
37	-0.171	-0.1	3.297	0.389408	-4.33276	37.12631
38	-0.324	1.309	3.377	-0.03838	-7.78885	39.5106
39	0.766	1.472	3.262	-0.53558	-4.39023	37.38343
40	-0.348	0.911	3.256	-2.41353	2.324757	35.83886

41	-0.323	0.196	3.325	-2.95579	6.564832	35.87714
42	-0.009	-0.152	3.48	-0.96621	7.913754	34.15304
43	0.158	-0.525	3.38	0.797193	7.56203	32.27519
44	0.623	-0.458	3.38	1.63654	6.66135	32.78142
45	-0.367	0.315	3.435	0.982799	4.077063	35.50027
46	0.273	0.926	3.382	0.545209	-0.03472	37.59753
47	-0.807	0.701	3.252	-0.05641	-3.79572	37.91593
48	0.52	1.149	3.374	0.853914	-4.66685	38.87671
49	-0.26	0.623	3.313	-0.85654	-0.47439	37.63473
50	-0.193	0.38	3.302	-0.91256	4.057626	34.9396
51	0.141	-0.144	3.188	0.073787	5.638878	31.74307
52	0.093	-0.272	3.238	1.070468	7.612053	30.05411
53	0.088	-0.082	3.495	0.928361	12.95655	28.70027
54	-0.107	0.309	3.545	-0.69018	14.50331	29.21238
55	-0.361	0.3	3.259	-1.7224	8.601415	32.44541
56	-0.234	0.179	3.255	-0.55591	1.172629	36.27057
57	0.427	0.697	3.358	0.705685	-6.14318	38.34962
58	0.022	1.072	3.708	0.890762	-9.08227	39.31689
59	0.635	0.638	3.679	0.643315	-7.73037	39.03275
60	-0.31	0.34	3.424	-1.06674	-1.47481	37.30523
61	-0.262	-0.086	3.326	-0.53141	3.613947	34.93667
62	0.062	0.027	3.395	-0.29163	6.830473	33.4069
63	-0.002	0.118	3.462	-0.83479	7.99815	32.36751
64	-0.333	-0.168	3.266	-0.74677	7.682884	31.91144
65	0.346	0.123	3.189	1.127941	6.32614	33.26209
66	0.466	0.758	3.23	1.798735	3.508645	36.67186
67	-0.686	1.396	3.499	-0.70008	-2.60951	39.65771
68	0.714	1.078	3.459	-0.5204	-5.85705	38.58421
69	-0.614	0.77	3.45	-0.12561	-5.04803	38.04811
70	0.373	1.206	3.413	0.562328	-1.30391	37.84212
71	0.327	0.548	3.221	-0.65025	4.099479	34.62278
72	-0.378	-0.082	3.316	-1.84169	6.330102	33.08315
73	-0.02	-0.592	3.33	-0.92631	6.681757	31.53013
74	0.02	-0.592	3.33	0.92631	6.681757	31.53013
75	0.378	-0.082	3.316	1.841688	6.330102	33.08315
76	-0.327	0.548	3.221	0.650248	4.099479	34.62278
77	-0.373	1.206	3.413	-0.56233	-1.30391	37.84212
78	0.614	0.77	3.45	0.125612	-5.04803	38.04811
79	-0.714	1.078	3.459	0.5204	-5.85705	38.58421
80	0.686	1.396	3.499	0.700083	-2.60951	39.65771
81	-0.466	0.758	3.23	-1.79874	3.508645	36.67186
82	-0.346	0.123	3.189	-1.12794	6.32614	33.26209
83	0.333	-0.168	3.266	0.74677	7.682884	31.91144
84	0.002	0.118	3.462	0.834793	7.99815	32.36751

85	-0.062	0.027	3.395	0.291625	6.830473	33.4069
86	0.262	-0.086	3.326	0.531411	3.613947	34.93667
87	0.31	0.34	3.424	1.06674	-1.47481	37.30523
88	-0.635	0.638	3.679	-0.64332	-7.73037	39.03275
89	-0.022	1.072	3.708	-0.89076	-9.08227	39.31689
90	-0.427	0.697	3.358	-0.70569	-6.14318	38.34962
91	0.234	0.179	3.255	0.555905	1.172629	36.27057
92	0.361	0.3	3.259	1.722396	8.601415	32.44541
93	0.107	0.309	3.545	0.690179	14.50331	29.21238
94	-0.088	-0.082	3.495	-0.92836	12.95655	28.70027
95	-0.093	-0.272	3.238	-1.07047	7.612053	30.05411
96	-0.141	-0.144	3.188	-0.07379	5.638878	31.74307
97	0.193	0.38	3.302	0.912564	4.057626	34.9396
98	0.26	0.623	3.313	0.856542	-0.47439	37.63473
99	-0.52	1.149	3.374	-0.85391	-4.66685	38.87671
100	0.807	0.701	3.252	0.056412	-3.79572	37.91593
101	-0.273	0.926	3.382	-0.54521	-0.03472	37.59753
102	0.367	0.315	3.435	-0.9828	4.077063	35.50027
103	-0.623	-0.458	3.38	-1.63654	6.66135	32.78142
104	-0.158	-0.525	3.38	-0.79719	7.56203	32.27519
105	0.009	-0.152	3.48	0.966208	7.913754	34.15304
106	0.323	0.196	3.325	2.955788	6.564832	35.87714
107	0.348	0.911	3.256	2.413526	2.324757	35.83886
108	-0.766	1.472	3.262	0.535575	-4.39023	37.38343
109	0.324	1.309	3.377	0.038384	-7.78885	39.5106
110	0.171	-0.1	3.297	-0.38941	-4.33276	37.12631
111	-0.056	-0.395	3.35	-0.21972	-1.6971	36.04724
112	-0.158	-0.466	3.393	0.010493	1.627476	35.27851
113	0.032	-0.419	3.357	-0.09059	5.583793	33.22854
114	-0.068	-0.184	3.34	-0.05027	8.675291	31.52951
115	-0.023	-0.079	3.352	-0.01813	7.966994	32.26486
116	0.201	-0.382	3.344	0.430487	4.633998	34.20234
117	-0.017	-0.408	3.519	0.261128	1.358497	36.56229
118	0.1	-0.019	3.353	1.098934	-2.78854	37.74678
119	-0.306	0.18	3.3	0.906138	-6.24982	37.94549
120	0.029	1.353	3.394	0.056972	-8.52332	40.37219
121	0.18	1.135	3.185	-0.99401	-3.51486	38.57783
122	-0.142	0.111	3.366	-0.0058	2.94471	35.63072
123	-0.063	0.119	3.502	1.456975	6.811052	34.73537
124	-0.031	-0.007	3.528	0.757466	9.781789	33.70512
125	0.075	-0.273	3.536	0.185494	7.94198	34.3279
126	0.019	-0.463	3.381	-0.55713	3.827899	35.00949
127	-0.022	-0.334	3.308	-0.92306	0.31391	35.96139
128	0.054	-0.309	3.404	-0.80282	-1.85335	36.71375

129	-0.216	0.214	3.387	-0.81116	-2.42103	37.27854
130	-0.717	1.495	3.371	-0.53306	-5.02049	39.6163
131	0.175	1.371	3.3	-0.80821	-3.7237	39.75333
132	-0.018	1.273	3.181	-1.93086	-0.25754	38.37625
133	0.222	0.954	3.461	-1.77318	5.857298	35.85245
134	-0.001	-0.178	3.385	-1.10858	9.720526	32.64112
135	-0.248	-0.564	3.355	-0.05323	8.456578	31.50373
136	0.282	-0.186	3.342	1.781782	7.115927	32.40929
137	0.203	0.17	3.266	0.781463	6.259892	33.49927
138	-0.235	0.034	3.303	-1.72573	2.590033	35.37039
139	-0.377	-0.424	3.344	-1.85369	-1.66689	36.37429
140	-0.011	-0.414	3.409	-0.90912	-2.73331	35.97841
141	-0.103	-0.166	3.314	-0.19035	-1.52692	35.01532
142	0.009	-0.225	3.194	1.204101	0.725613	34.68018
143	0.333	0.367	3.356	1.362311	3.860232	35.58183
144	-0.04	0.189	3.344	-0.39818	4.495718	35.9233
145	-0.011	-0.478	3.252	-2.11259	1.872463	36.26898
146	NA	NA	NA	NA	NA	NA

Note: the PDB codes of the nucleosomes used to obtain the template structure listed in this table are: 1EQZ, 1KX5, 1P3A, 1P3B, 1P3F, 1P3G, 1P3I, 1U35, 2CV5, 2NZD, 2PYO, 3AFA, 3AV1, 3AV2, 3AYW, 3AZF, 3AZG, 3AZI, 3AZJ, 3AZL, 3AZM, 3AZN, 3LEL, 3LZ0, 3LZ1, 3REH, 3REI, 3REJ, 3REK, 3REL, 3UT9, 3UTA, , 3UTB, 3X1V, 4Z5T, 5AV5, 5AV6, 5AV8, 5AV9, 5AVB, 5AVC, 5AY8, 5B0Y, 5B0Z, 5B1L, 5B1M, 5B31, 5B32, 5F99, 5GXQ, 5X7X, 5Y0C and 5Y0D. These base-step parameters are symmetric relative to nucleosome dyad position (shift and tilt are anti-symmetric).

Table S6. Position-dependent parameters of the template nucleosome structure obtained by averaging over 19 nucleosomes whose nucleosomal DNA length is 146bp

Step position	Shift	Slide	Rise	Tilt	Roll	Twist
1	-0.164	-0.719	3.167	1.332641	0.195388	35.3437
2	0.166	-0.178	3.13	2.022714	3.029578	35.99323
3	-0.337	0.471	3.391	-0.45305	4.803414	36.11116
4	0.07	0.069	3.269	-1.4077	2.589448	35.37281
5	-0.017	-0.548	3.141	-0.23101	0.044211	32.88095
6	0.132	0.222	3.359	0.616515	-2.0849	35.6624
7	-0.038	-0.683	3.415	0.875227	-2.71807	36.25389
8	0.533	-0.158	3.318	1.771556	-0.52585	36.46006
9	0.004	0.07	3.161	0.670964	3.808219	34.21545
10	-0.507	0.151	3.352	-2.39921	6.65497	33.20028
11	-0.049	-0.777	3.225	-1.07444	7.152743	31.47705
12	0.508	-0.532	3.488	0.934806	11.01912	31.21288
13	-0.454	0.499	3.24	1.517306	8.497579	33.70731
14	0.05	1.616	3.462	1.309703	1.270933	38.1406
15	-0.246	0.666	3.036	2.054541	-2.91556	38.86041
16	0.14	2.068	3.579	1.37888	-5.04777	41.16569
17	0.757	0.717	3.34	1.002163	-2.01494	38.05398
18	-0.493	-0.038	3.441	0.583994	-0.69992	37.39204
19	0.176	-0.302	3.285	0.335194	-0.84345	36.1002
20	-0.146	-0.181	3.219	0.397087	2.099377	35.17979
21	0.173	-0.52	3.485	0.053214	6.514944	34.5527
22	-0.281	-0.038	3.459	-0.49451	9.798138	33.49272
23	0.336	0.39	3.483	-1.68383	9.785909	33.13169
24	-0.25	0.105	3.392	-1.21547	5.253595	33.30504
25	0.422	0.22	3.075	1.67881	0.666979	36.12591
26	-0.501	2.299	3.299	0.490627	-6.58256	41.34528
27	0.571	0.41	3.376	-1.0935	-6.76006	39.00323
28	-0.124	-0.031	3.297	-1.60529	-4.37714	37.07043
29	-0.023	-0.25	3.329	-0.42591	-1.0775	36.52211
30	-0.009	-0.329	3.546	-0.31673	3.442325	35.03036
31	-0.278	-0.514	3.228	-0.73512	6.329547	33.37795
32	0.183	0.223	3.612	-0.70357	8.786853	33.35937
33	-0.019	-0.447	3.116	-0.62038	6.689884	31.46728
34	-0.02	-0.364	3.452	0.932262	3.860014	34.31675
35	0.233	-0.565	3.331	0.961951	0.711515	34.44045
36	-0.009	-0.191	3.343	0.354339	-2.31052	35.94698
37	-0.225	0.337	3.321	-0.37453	-5.52083	38.73065
38	-0.109	1.893	3.4	-0.31827	-7.84304	40.03373
39	0.907	0.974	3.144	-0.11397	-0.48645	35.83056
40	-0.926	1.01	3.391	-2.68985	5.376809	35.44831

41	0.018	-0.039	3.289	-3.08418	7.516159	34.95522
42	-0.029	-0.254	3.811	-1.32638	7.869598	34.90607
43	0.218	-0.893	3.252	0.863489	6.942567	31.69569
44	0.605	-0.368	3.403	3.153222	6.128325	33.22218
45	-0.487	1.036	3.426	0.791911	2.759351	36.70507
46	0.146	0.531	3.31	-1.21878	-2.12871	37.74314
47	-0.746	1.047	3.261	0.363171	-5.97174	38.31827
48	0.75	0.932	3.336	1.300885	-3.82627	38.96346
49	-0.676	0.367	3.232	-0.99671	2.055525	36.11119
50	0.177	0.438	3.418	-0.28119	5.619394	33.84653
51	0.144	-0.439	2.997	0.685603	4.977752	30.28649
52	0.267	0.002	3.289	1.849111	9.713737	29.54382
53	-0.226	-0.031	3.75	-0.47122	15.89678	28.75583
54	-0.07	0.317	3.374	-1.66519	12.29959	30.69147
55	-0.421	0.248	3.187	-1.30463	6.187716	34.4348
56	-0.12	0.36	3.272	-0.41396	-2.52456	37.17617
57	0.523	1.104	3.417	-0.01062	-8.5573	38.35916
58	0.011	1.064	3.754	0.401602	-10.3703	39.2879
59	0.438	0.283	3.541	1.165686	-4.85591	38.37649
60	-0.51	0.516	3.55	-0.8036	2.547604	35.84467
61	-0.005	-0.583	3.229	-1.2741	5.542837	32.7919
62	0.151	0.501	3.671	-0.99396	7.649415	33.14955
63	-0.311	-0.436	3.219	-1.22743	6.885914	32.09521
64	-0.042	-0.046	3.258	0.637763	6.557795	32.56253
65	0.76	0.311	3.17	2.085287	5.002725	34.88573
66	-0.358	1.341	3.414	1.25144	0.909645	38.08943
67	0.016	1.079	3.319	-1.3565	-3.84101	38.93347
68	0.142	0.927	3.595	-0.57308	-5.75225	38.72729
69	-0.284	1.084	3.456	0.593118	-2.93563	38.11493
70	0.534	1.137	3.257	-0.43643	1.971257	36.22005
71	-0.382	0.233	3.283	-2.0312	5.325104	34.14626
72	-0.073	-0.294	3.298	-1.54243	6.811842	32.47603
73	0	-0.839	3.32	-0.07485	7.117132	29.01363
74	0.073	-0.294	3.298	0.074851	7.117132	29.01363
75	0.382	0.233	3.283	1.542434	6.811842	32.47603
76	-0.534	1.137	3.257	2.031203	5.325104	34.14626
77	0.284	1.084	3.456	0.436433	1.971257	36.22005
78	-0.142	0.927	3.595	-0.59312	-2.93563	38.11493
79	-0.016	1.079	3.319	0.57308	-5.75225	38.72729
80	0.358	1.341	3.414	1.3565	-3.84101	38.93347
81	-0.76	0.311	3.17	-1.25144	0.909645	38.08943
82	0.042	-0.046	3.258	-2.08529	5.002725	34.88573
83	0.311	-0.436	3.219	-0.63776	6.557795	32.56253
84	-0.151	0.501	3.671	1.227431	6.885914	32.09521

85	0.005	-0.583	3.229	0.993957	7.649415	33.14955
86	0.51	0.516	3.55	1.274095	5.542837	32.7919
87	-0.438	0.283	3.541	0.803597	2.547604	35.84467
88	-0.011	1.064	3.754	-1.16569	-4.85591	38.37649
89	-0.523	1.104	3.417	-0.4016	-10.3703	39.2879
90	0.12	0.36	3.272	0.010621	-8.5573	38.35916
91	0.421	0.248	3.187	0.413961	-2.52456	37.17617
92	0.07	0.317	3.374	1.304628	6.187716	34.4348
93	0.226	-0.031	3.75	1.66519	12.29959	30.69147
94	-0.267	0.002	3.289	0.471223	15.89678	28.75583
95	-0.144	-0.439	2.997	-1.84911	9.713737	29.54382
96	-0.177	0.438	3.419	-0.6856	4.977752	30.28649
97	0.676	0.367	3.232	0.281189	5.619394	33.84653
98	-0.75	0.932	3.336	0.996711	2.055525	36.11119
99	0.746	1.047	3.261	-1.30089	-3.82627	38.96346
100	-0.146	0.531	3.311	-0.36317	-5.97174	38.31827
101	0.487	1.036	3.426	1.218783	-2.12871	37.74314
102	-0.606	-0.368	3.403	-0.79191	2.759351	36.70507
103	-0.218	-0.893	3.252	-3.15322	6.128325	33.22218
104	0.029	-0.254	3.811	-0.86349	6.942567	31.69569
105	-0.018	-0.039	3.289	1.326382	7.869598	34.90607
106	0.926	1.01	3.391	3.084175	7.516159	34.95522
107	-0.907	0.974	3.144	2.68985	5.376809	35.44831
108	0.109	1.893	3.4	0.113966	-0.48645	35.83056
109	0.225	0.337	3.321	0.318268	-7.84304	40.03373
110	0.009	-0.191	3.343	0.374526	-5.52083	38.73065
111	-0.233	-0.565	3.331	-0.35434	-2.31052	35.94698
112	0.02	-0.364	3.452	-0.96195	0.711515	34.44045
113	0.019	-0.447	3.116	-0.93226	3.860014	34.31675
114	-0.183	0.223	3.612	0.620383	6.689884	31.46728
115	0.278	-0.514	3.228	0.703566	8.786853	33.35937
116	0.009	-0.329	3.546	0.73512	6.329547	33.37795
117	0.023	-0.25	3.329	0.316727	3.442325	35.03036
118	0.124	-0.031	3.297	0.425909	-1.0775	36.52211
119	-0.571	0.41	3.376	1.605286	-4.37714	37.07043
120	0.501	2.299	3.299	1.093504	-6.76006	39.00323
121	-0.422	0.22	3.075	-0.49063	-6.58256	41.34528
122	0.25	0.105	3.392	-1.67881	0.666979	36.12591
123	-0.336	0.39	3.483	1.215472	5.253595	33.30504
124	0.281	-0.038	3.459	1.683832	9.785909	33.13169
125	-0.173	-0.52	3.485	0.494513	9.798138	33.49272
126	0.146	-0.181	3.219	-0.05321	6.514944	34.5527
127	-0.176	-0.302	3.285	-0.39709	2.099377	35.17979
128	0.493	-0.038	3.441	-0.33519	-0.84345	36.1002

129	-0.757	0.717	3.34	-0.58399	-0.69992	37.39204
130	-0.14	2.068	3.579	-1.00216	-2.01494	38.05398
131	0.246	0.666	3.036	-1.37888	-5.04777	41.16569
132	-0.05	1.616	3.462	-2.05454	-2.91556	38.86041
133	0.454	0.499	3.24	-1.3097	1.270933	38.1406
134	-0.508	-0.532	3.488	-1.51731	8.497579	33.70731
135	0.049	-0.777	3.225	-0.93481	11.01912	31.21288
136	0.507	0.151	3.352	1.074441	7.152743	31.47705
137	-0.004	0.07	3.161	2.399213	6.65497	33.20028
138	-0.533	-0.158	3.318	-0.67096	3.808219	34.21545
139	0.038	-0.683	3.415	-1.77156	-0.52585	36.46006
140	-0.132	0.222	3.359	-0.87523	-2.71807	36.25389
141	0.017	-0.548	3.141	-0.61652	-2.0849	35.6624
142	-0.07	0.069	3.269	0.231011	0.044211	32.88095
143	0.337	0.471	3.391	1.407698	2.589448	35.37281
144	-0.166	-0.178	3.13	0.453049	4.803414	36.11116
145	0.164	-0.719	3.167	-2.02271	3.029578	35.99323

Note: the PDB codes of the nucleosomes used to obtain the template structure listed in this table are: 1EQZ, 1P3A, 1P3B, 1P3F, 1P3G, 1P3I, 2CV5, 3AFA, 3AV1, 3AV2, 3REJ, 3REK, 3REL, 3UTB, 4Z5T, 5B0Y, 5B0Z, 5B1L and 5Y0C. These base-step parameters are symmetric relative to nucleosome dyad position (shift and tilt are anti-symmetric).

Table S7. Position-dependent parameters of the template nucleosome structure obtained by averaging over 24 nucleosomes whose nucleosomal DNA length is 145bp

Step position	Shift	Slide	Rise	Tilt	Roll	Twist
1	-0.296	-0.703	3.296	1.922414	2.089053	36.44405
2	0.394	0.189	3.381	1.513158	4.981316	36.45635
3	-0.444	0.527	3.391	-1.13806	5.447129	35.5422
4	-0.155	-0.042	3.195	-2.36405	1.891952	35.17121
5	0.127	-0.198	3.217	-0.18048	-0.94243	34.40043
6	-0.047	-0.405	3.508	1.126651	-2.938	35.79925
7	0.342	-0.496	3.356	1.989296	-2.26106	36.91479
8	0.488	-0.026	3.404	2.261288	2.270371	36.21037
9	-0.115	0.256	3.27	-0.49572	6.673516	33.5192
10	-0.611	0.17	3.358	-2.81568	7.102201	32.32028
11	0.144	-0.544	3.304	-0.65459	7.506294	31.34898
12	0.202	-0.518	3.461	0.776562	11.22724	32.37626
13	-0.44	0.644	3.548	1.981028	9.115052	35.3289
14	0.204	1.749	3.278	2.043527	0.710292	39.11081
15	-0.531	1.032	3.229	0.358981	-2.57959	39.40585
16	0.905	2.172	3.305	0.330135	-7.18608	40.57387
17	0.502	0.385	3.35	0.933849	-3.25947	37.51111
18	-0.141	-0.257	3.465	1.297935	-2.398	37.34262
19	0.162	-0.314	3.407	1.145002	-1.28691	37.75409
20	0.096	-0.419	3.425	0.640775	2.019555	36.7977
21	-0.129	-0.357	3.609	-0.4431	6.809319	35.82158
22	-0.12	0.021	3.594	-1.03747	8.997348	34.71669
23	0.096	0.242	3.496	-1.97359	5.782338	35.98591
24	0.105	0.107	3.612	-1.16732	2.59941	37.10521
25	-0.258	0.621	3.151	1.155735	-3.41748	38.69532
26	-0.145	1.746	3.436	0.450201	-10.4791	41.25983
27	0.318	0.349	3.233	-1.02803	-7.17132	38.44383
28	-0.204	0.101	3.378	-1.47834	-3.33888	39.60119
29	0.059	-0.433	3.602	-0.21268	1.205852	38.23929
30	-0.159	-0.375	3.388	-0.60339	4.607511	34.38621
31	-0.067	0.002	3.253	0.534301	8.136681	31.66918
32	0.188	-0.121	3.395	0.277097	9.434586	30.77501
33	-0.066	-0.434	3.303	-0.14017	5.437714	33.25461
34	0.139	-0.404	3.429	-0.27994	1.259441	35.85764
35	0.095	-0.491	3.301	0.2361	-1.87168	36.50061
36	-0.228	-0.205	3.302	0.985012	-4.0813	36.79786
37	-0.345	1.128	3.357	0.404645	-7.43666	39.58627
38	0.871	1.631	3.337	-0.72534	-4.95317	37.8893
39	-0.249	0.92	3.231	-3.31766	2.072485	36.17207
40	-0.376	0.081	3.312	-3.34497	6.859337	36.35936

41	-0.048	-0.17	3.474	-0.12497	8.149274	34.34458
42	0.075	-0.469	3.325	0.731438	7.516025	31.92495
43	0.618	-0.549	3.485	0.498958	6.283625	32.79879
44	-0.357	0.125	3.455	0.899692	3.809613	35.50263
45	0.37	1.027	3.353	1.569297	0.394822	37.60743
46	-0.959	0.702	3.218	0.203895	-3.38976	38.24453
47	0.501	1.297	3.426	0.610946	-4.60487	39.12248
48	-0.121	0.675	3.322	-1.40882	0.038422	37.78725
49	-0.237	0.358	3.268	-1.47296	4.42673	34.95783
50	0.045	-0.096	3.241	-0.5581	6.605353	31.69468
51	-0.025	-0.384	3.287	0.253803	7.80905	30.13047
52	0.233	-0.173	3.445	0.649599	12.03229	29.11429
53	-0.237	0.366	3.645	-0.56992	14.37179	29.58649
54	-0.365	0.325	3.271	-1.87737	8.019228	32.99426
55	-0.211	0.115	3.233	-0.62573	0.476774	36.6935
56	0.461	0.585	3.4	1.380243	-6.31311	39.02859
57	0.075	0.976	3.72	1.641331	-8.5813	39.26511
58	0.731	0.631	3.671	0.295961	-7.95717	39.05967
59	-0.294	0.227	3.326	-1.75323	-2.31493	37.96784
60	-0.287	0.034	3.282	0.233476	3.364123	36.03625
61	0.014	-0.067	3.321	0.028517	7.000825	33.57209
62	0.098	0.316	3.616	-0.88746	8.984437	32.26115
63	-0.44	-0.179	3.239	-1.21093	8.259934	31.48711
64	0.258	0.088	3.146	0.887012	6.308025	32.56141
65	0.68	0.717	3.18	1.853537	3.391127	36.7775
66	-1	1.552	3.581	-0.90817	-3.07858	40.47698
67	1.063	1.066	3.427	-0.4609	-5.98085	38.34041
68	-0.876	0.672	3.438	-0.29691	-5.54537	38.17662
69	0.41	1.216	3.451	1.09507	-1.7433	38.18202
70	0.558	0.579	3.25	0.116546	4.613595	34.37972
71	-0.479	-0.039	3.299	-1.97686	6.84996	32.98465
72	0.027	-0.581	3.346	-0.7471	7.167388	31.41046
73	-0.027	-0.581	3.346	0.747097	7.167388	31.41046
74	0.479	-0.039	3.299	1.976855	6.84996	32.98465
75	-0.557	0.579	3.25	-0.11655	4.613595	34.37972
76	-0.41	1.216	3.451	-1.09507	-1.7433	38.18202
77	0.876	0.672	3.438	0.296913	-5.54537	38.17662
78	-1.063	1.066	3.427	0.460901	-5.98085	38.34041
79	1	1.552	3.581	0.908167	-3.07858	40.47698
80	-0.68	0.717	3.18	-1.85354	3.391127	36.7775
81	-0.258	0.088	3.146	-0.88701	6.308025	32.56141
82	0.44	-0.179	3.239	1.210931	8.259934	31.48711
83	-0.098	0.316	3.616	0.88746	8.984437	32.26115
84	-0.014	-0.067	3.321	-0.02852	7.000825	33.57209

85	0.287	0.034	3.282	-0.23348	3.364123	36.03625
86	0.294	0.227	3.326	1.753229	-2.31493	37.96784
87	-0.731	0.631	3.671	-0.29596	-7.95717	39.05967
88	-0.075	0.976	3.72	-1.64133	-8.5813	39.26511
89	-0.461	0.585	3.4	-1.38024	-6.31311	39.02859
90	0.211	0.115	3.233	0.625728	0.476774	36.6935
91	0.365	0.325	3.271	1.877371	8.019228	32.99426
92	0.237	0.366	3.645	0.569917	14.37179	29.58649
93	-0.233	-0.173	3.445	-0.6496	12.03229	29.11429
94	0.025	-0.384	3.287	-0.2538	7.80905	30.13047
95	-0.045	-0.096	3.241	0.558098	6.605353	31.69468
96	0.237	0.358	3.268	1.472962	4.42673	34.95783
97	0.121	0.675	3.322	1.40882	0.038422	37.78725
98	-0.501	1.297	3.426	-0.61095	-4.60487	39.12248
99	0.959	0.702	3.218	-0.2039	-3.38976	38.24453
100	-0.37	1.027	3.353	-1.5693	0.394822	37.60743
101	0.357	0.125	3.455	-0.89969	3.809613	35.50263
102	-0.618	-0.549	3.485	-0.49896	6.283625	32.79879
103	-0.075	-0.469	3.325	-0.73144	7.516025	31.92495
104	0.048	-0.17	3.474	0.124969	8.149274	34.34458
105	0.376	0.081	3.312	3.344973	6.859337	36.35936
106	0.249	0.92	3.231	3.317663	2.072485	36.17207
107	-0.871	1.631	3.337	0.72534	-4.95317	37.8893
108	0.345	1.128	3.357	-0.40465	-7.43666	39.58627
109	0.228	-0.205	3.302	-0.98501	-4.0813	36.79786
110	-0.095	-0.491	3.301	-0.2361	-1.87168	36.50061
111	-0.139	-0.404	3.429	0.27994	1.259441	35.85764
112	0.066	-0.434	3.303	0.140167	5.437714	33.25461
113	-0.188	-0.121	3.395	-0.2771	9.434586	30.77501
114	0.067	0.002	3.253	-0.5343	8.136681	31.66918
115	0.159	-0.375	3.388	0.603391	4.607511	34.38621
116	-0.059	-0.433	3.602	0.212675	1.205852	38.23929
117	0.204	0.101	3.378	1.47834	-3.33888	39.60119
118	-0.318	0.349	3.233	1.028034	-7.17132	38.44383
119	0.145	1.746	3.436	-0.4502	-10.4791	41.25983
120	0.258	0.621	3.151	-1.15574	-3.41748	38.69532
121	-0.105	0.107	3.612	1.167323	2.59941	37.10521
122	-0.096	0.242	3.496	1.973594	5.782338	35.98591
123	0.12	0.021	3.594	1.037473	8.997348	34.71669
124	0.129	-0.357	3.609	0.443096	6.809319	35.82158
125	-0.096	-0.419	3.425	-0.64078	2.019555	36.7977
126	-0.162	-0.314	3.407	-1.145	-1.28691	37.75409
127	0.141	-0.257	3.465	-1.29794	-2.398	37.34262
128	-0.502	0.385	3.35	-0.93385	-3.25947	37.51111

129	-0.905	2.172	3.305	-0.33014	-7.18608	40.57387
130	0.531	1.032	3.229	-0.35898	-2.57959	39.40585
131	-0.204	1.749	3.278	-2.04353	0.710292	39.11081
132	0.44	0.644	3.548	-1.98103	9.115052	35.3289
133	-0.202	-0.518	3.461	-0.77656	11.22724	32.37626
134	-0.144	-0.544	3.304	0.654591	7.506294	31.34898
135	0.611	0.17	3.358	2.815678	7.102201	32.32028
136	0.115	0.256	3.27	0.495723	6.673516	33.5192
137	-0.488	-0.026	3.404	-2.26129	2.270371	36.21037
138	-0.342	-0.496	3.356	-1.9893	-2.26106	36.91479
139	0.047	-0.405	3.508	-1.12665	-2.938	35.79925
140	-0.127	-0.198	3.217	0.180479	-0.94243	34.40043
141	0.155	-0.042	3.195	2.364054	1.891952	35.17121
142	0.444	0.527	3.391	1.138056	5.447129	35.5422
143	-0.394	0.189	3.381	-1.51316	4.981316	36.45635
144	0.296	-0.703	3.296	-1.92241	2.089053	36.44405

Note: the PDB codes of the nucleosomes used to obtain the template structure listed in this table are: 1U35, 2NZD, 3AYW, 3AZF, 3AZG, 3AZI, 3AZJ, 3AZL, 3AZM, 3AZN, 3LZ0, 3LZ1, 3REH, 3REI, 3UT9, 3UTA, , 3X1V, 5AY8, 5B1M, 5B31, 5B32, 5GXQ, 5X7X and 5Y0D. These base-step parameters are symmetric relative to nucleosome dyad position (shift and tilt are anti-symmetric).

Table S8. Pearson correlations of nucleosome occupancy predicted based on different template nucleosome structures with experimental nucleosome map

yeast	Template (145-147bp)	Template (146bp)	Template (145bp)
chrI	**0.500**	**0.516**	**0.512**
chrII	**0.430**	**0.449**	**0.444**
chrIII	**0.552**	**0.570**	**0.566**
chrIV	**0.430**	**0.447**	**0.443**
chrV	**0.447**	**0.467**	**0.462**
chrVI	**0.447**	**0.465**	**0.461**
chrVII	**0.422**	**0.440**	**0.436**
chrVIII	**0.419**	**0.438**	**0.433**
chrIX	**0.494**	**0.512**	**0.508**
chrX	**0.467**	**0.485**	**0.480**
chrXI	**0.443**	**0.459**	**0.455**
chrXII	**0.135**	**0.139**	**0.138**
chrXIII	**0.426**	**0.444**	**0.439**
chrXIV	**0.432**	**0.450**	**0.446**
chrXV	**0.430**	**0.448**	**0.443**
chrXVI	**0.435**	**0.453**	**0.448**

Note: significant correlations at the level $P<0.0001$ are shown in bold. MNase-digestion map (400U IP map from Chereji et al. 2017) was used in the correlation analysis. The three template nucleosome structures used in deformation energy calculation refer to Table S5, Table S6 and Table S7.

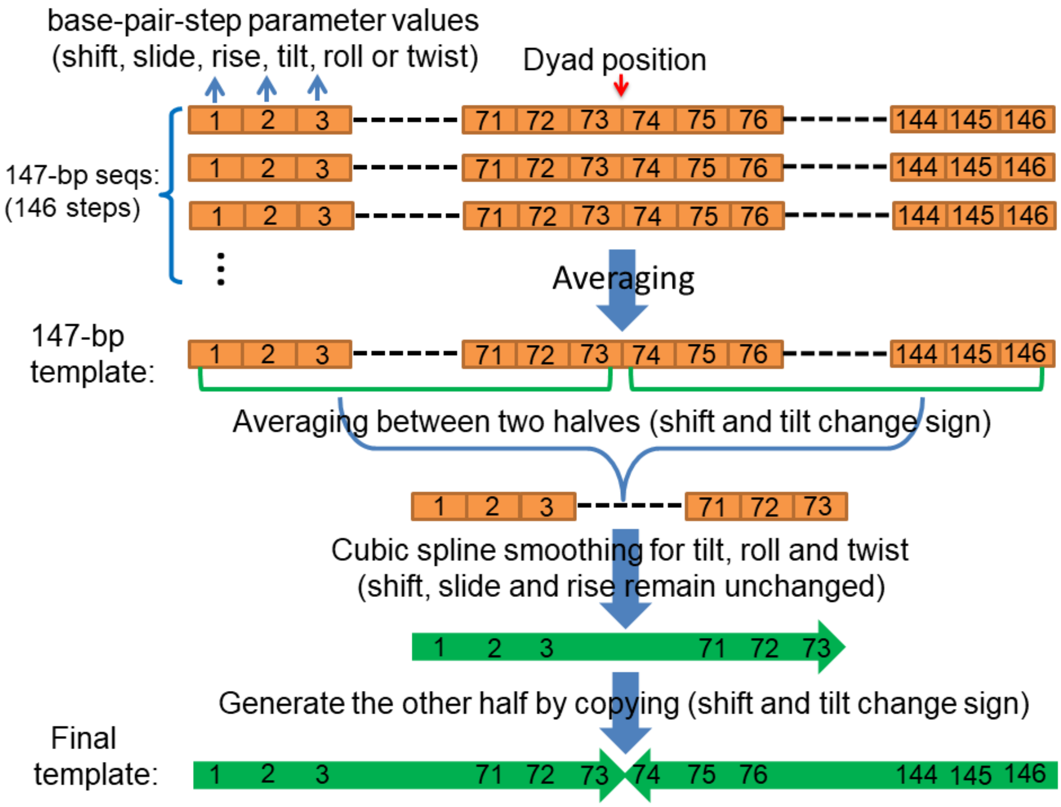

Figure S1. The averaging process used to obtain template nucleosome structure listed in Table S1. Six nucleosomal DNA structures from PDB (5AV6, 5AV8, 5AV9, 5AVB, 5OMX, 5ONW) are aligned at the dyad position, and then averaged for each of the six base-pair-step parameters (shift, slide, rise, tilt, roll and twist). In order to obtain a symmetric template, two halves of the previous-step structure are averaged relative to the dyad position (the signs of tilt and shift for the half of the structure need to be changed manually before averaging in this step). After averaging the two halves, obtained rotational parameters (tilt, roll and twist) is smoothed by using a cubic spline fit to reduce the noise in their 10-bp periodical oscillation. Translational parameters (shift, slide and rise) do not show strong oscillation pattern and therefore are not smoothed. Finally, final template structure is obtained by copying and concatenating (shift and tilt also change signs in the duplicated copy). The final template structure obtained here ensures that DNA deformation energy calculated for Watson strand is the same with that of Crick strand.

Figure S2

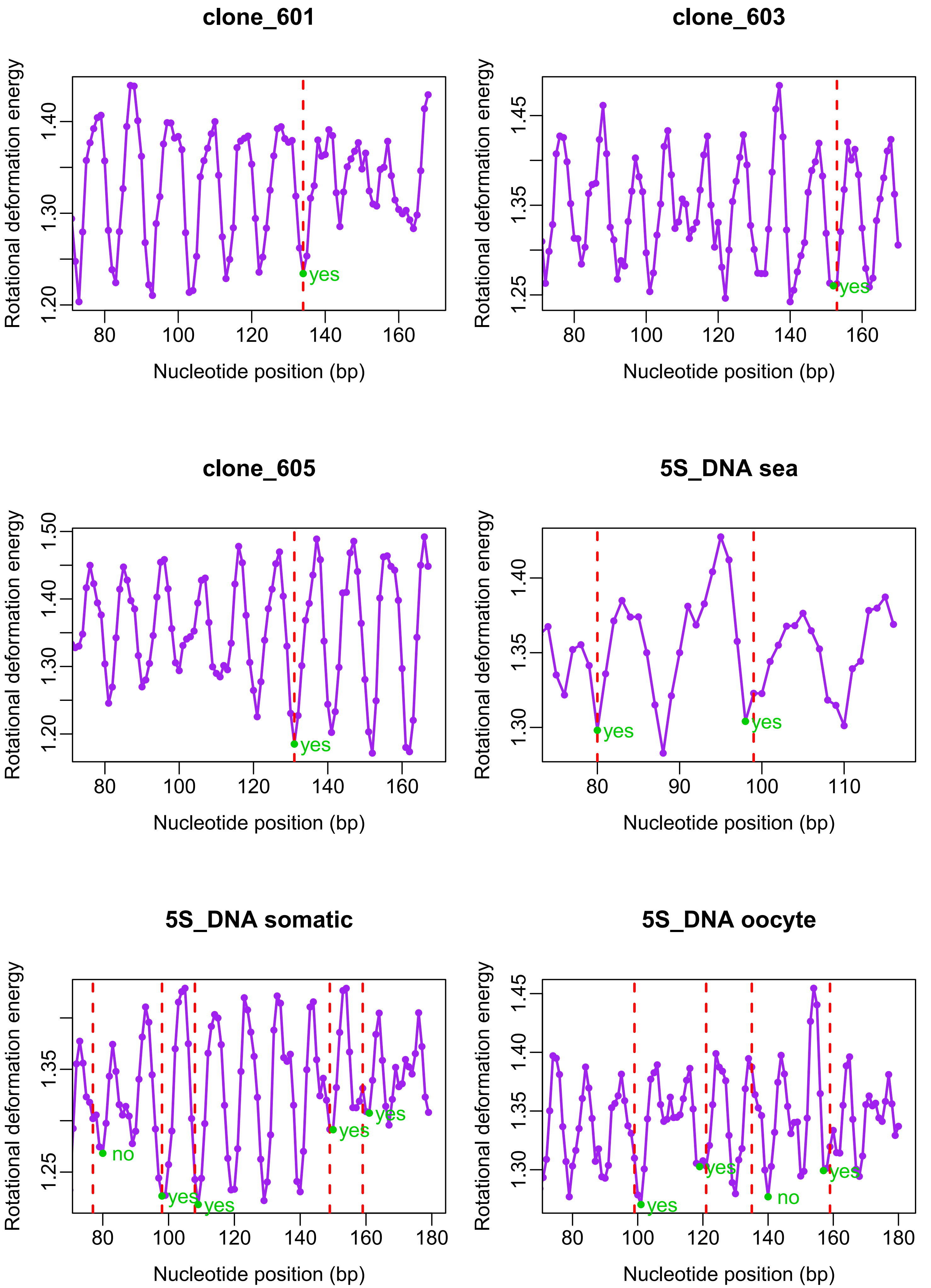

clone_601
clone_603
clone_605
5S_DNA sea
5S_DNA somatic
5S_DNA oocyte
Rotational deformation energy
Nucleotide position (bp)
yes
no

Figure S2 (continued).

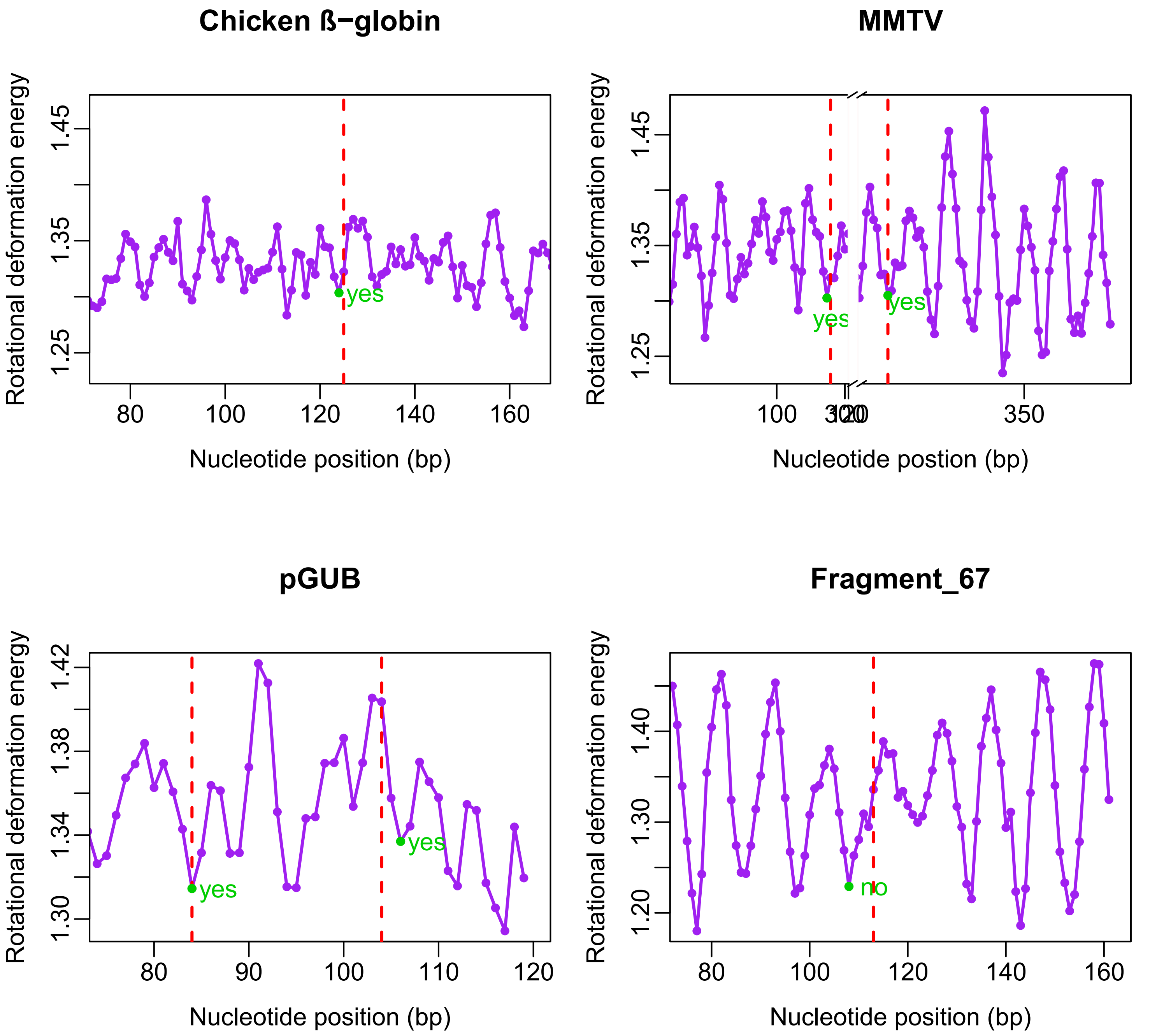

Figure S2. Prediction of rotational positioning of 20 nucleosomes assembled in vitro. Small rotational deformation energy indicates a high probability of placing the dyad of a nucleosome at that position, where the major groove of DNA faces the histones. It is marked as "yes" if the discrepancy between the local minimum of rotational deformation energy (green solid ball) and the experimentally determined dyad positions (vertical dash line) is no more than 2 bp, otherwise, marked as "no". The local energy minimum is defined as the smallest value in an 11-bp span centering at the dyad position and such a span is adequate to capture the most probable rotational position within the range of one DNA helical turn.

Figure S3

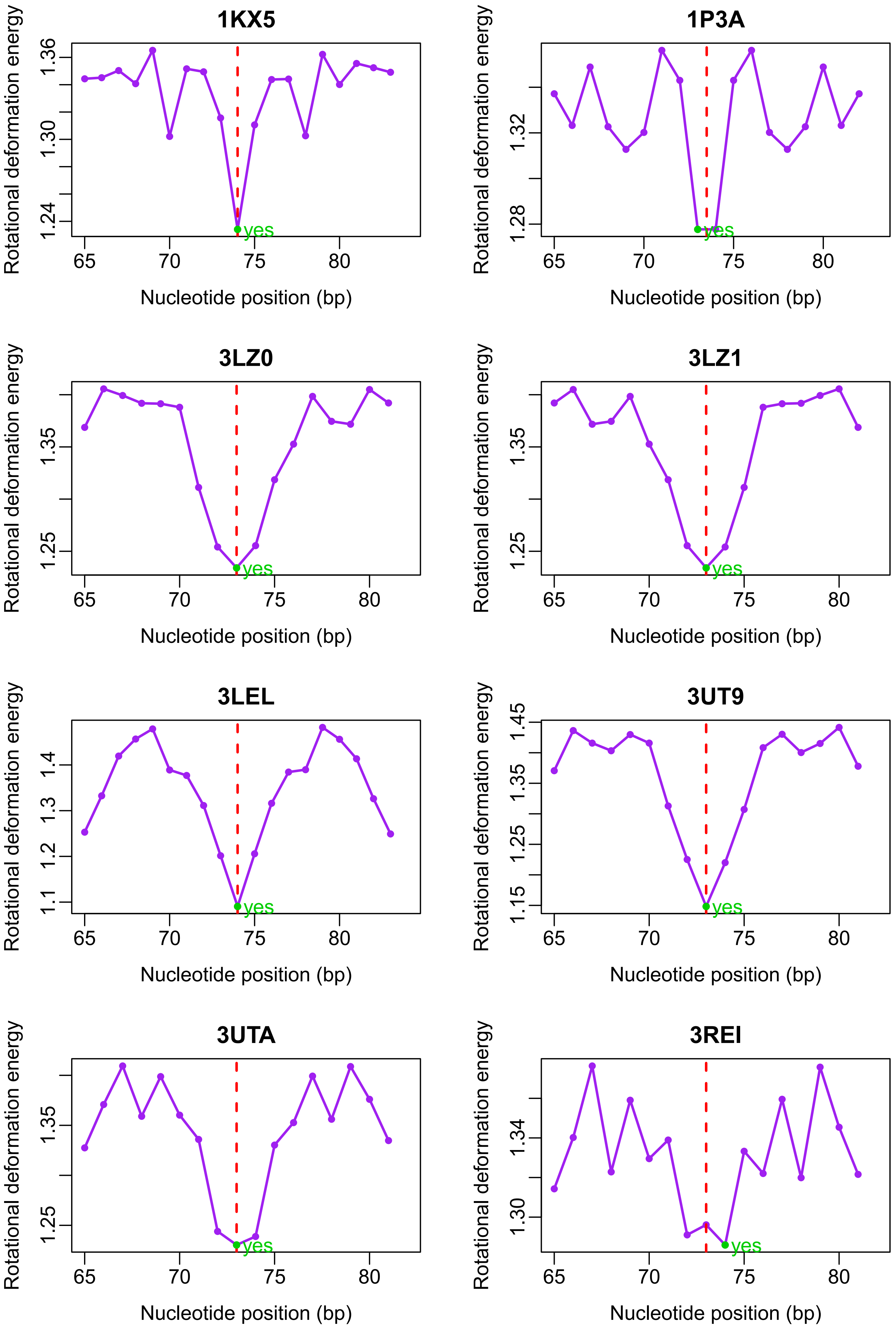
1KX5
1P3A
3LZ0
3LZ1
3LEL
3UT9
3UTA
3REI
Rotational deformation energy
Nucleotide position (bp)
yes
1.24
1.30
1.36
1.28
1.32
1.25
1.35
1.1
1.2
1.3
1.4
1.15
1.45
65
70
75
80

Figure S3 (continued).

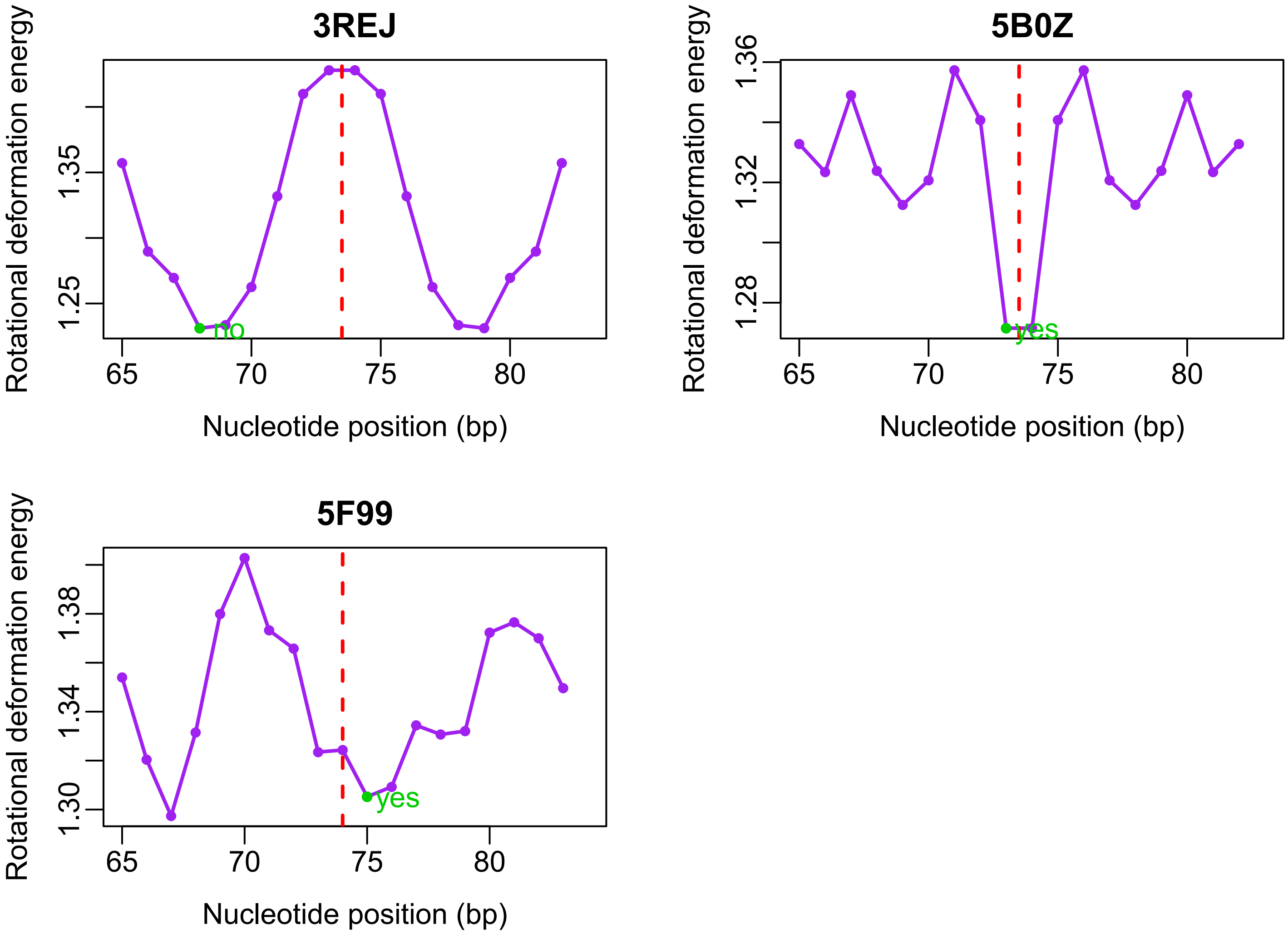

Figure S3. Prediction of rotational positioning of nucleosomes taken from PDB. Legends are the same as in Figure S2.

Figure S4 (fold 1)

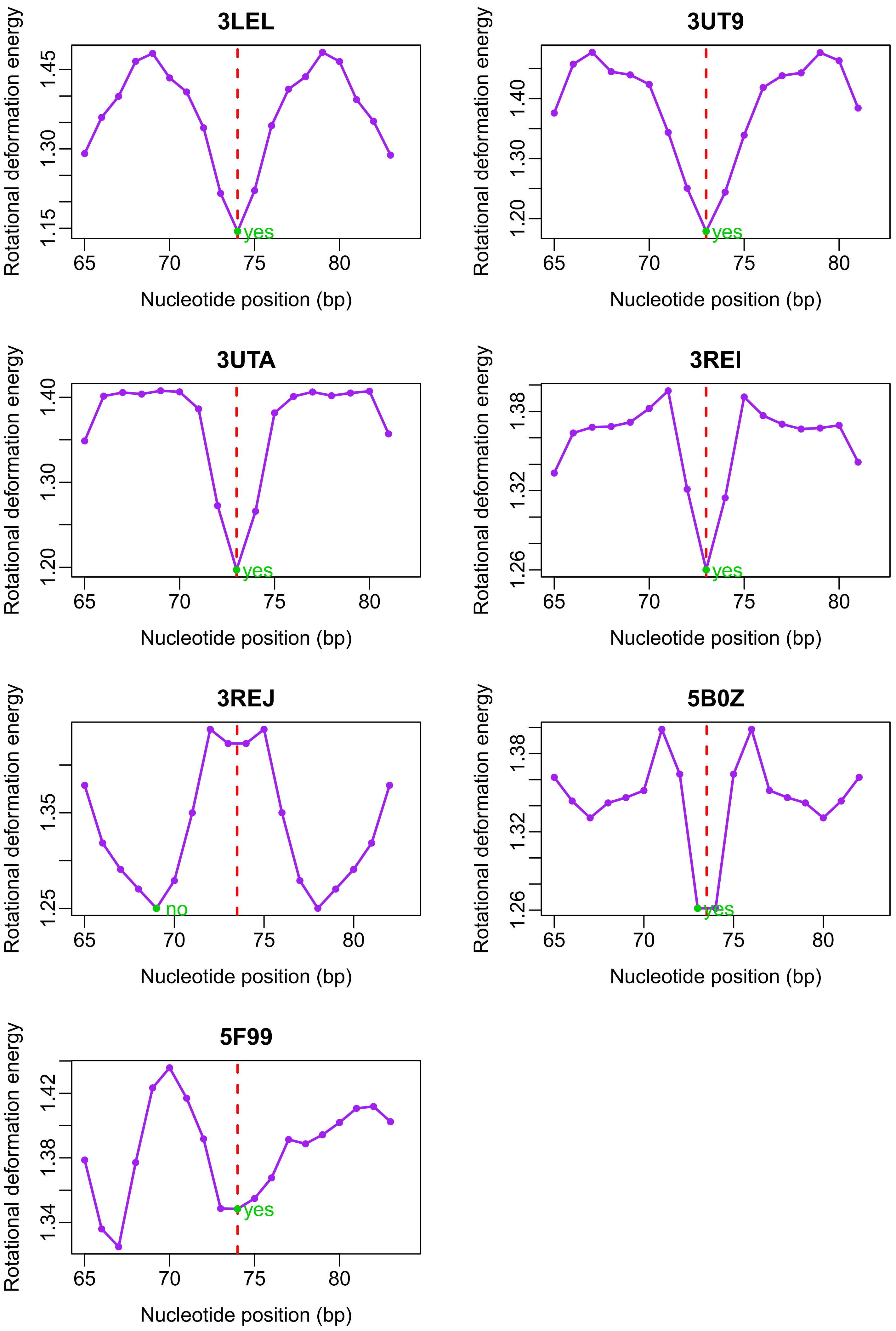

Figure S4 (fold 2)

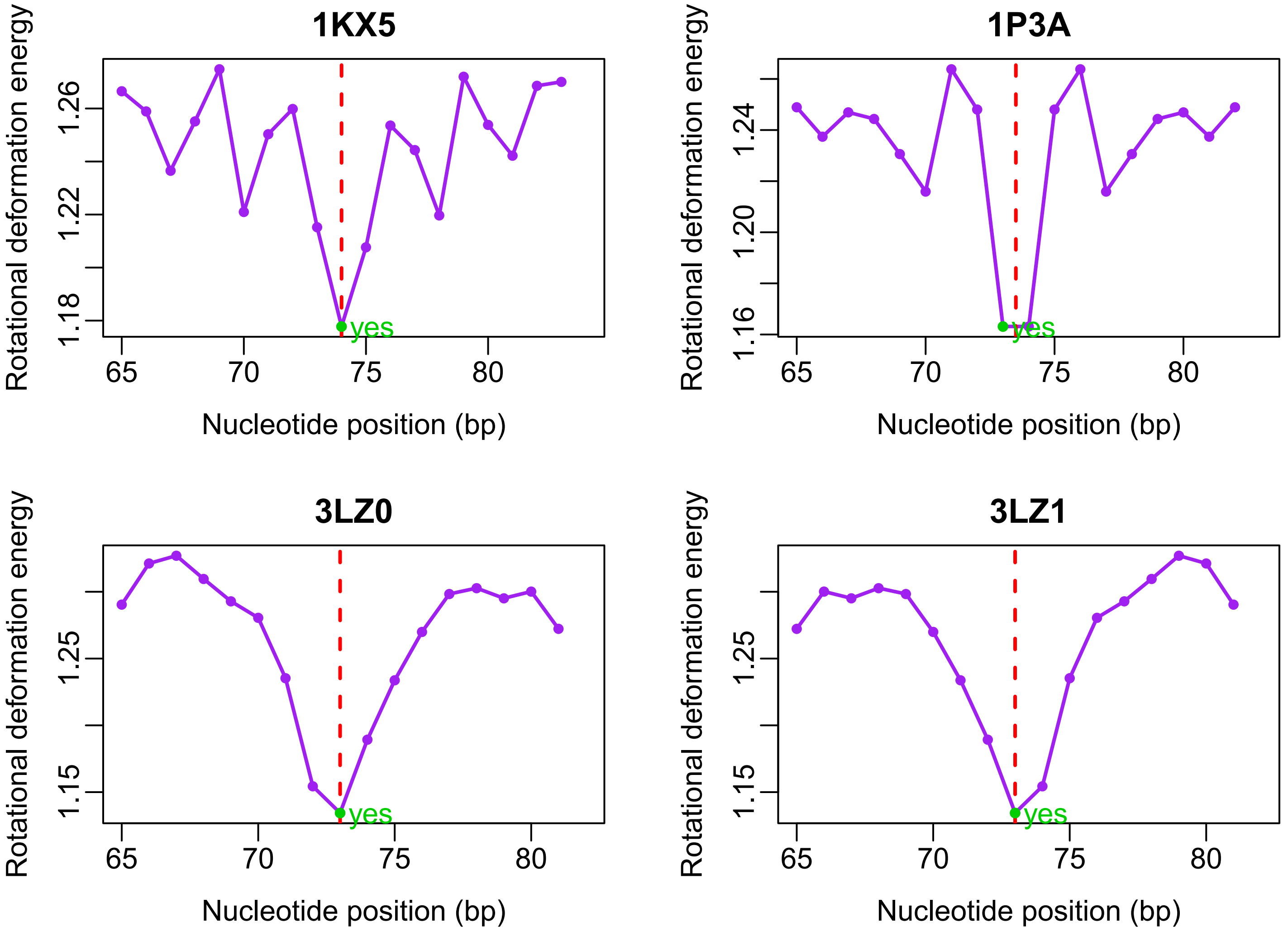

Figure S4. 2-Fold prediction of rotational positioning of nucleosomes taken from PDB. We randomly separated the most diverse nucleosome reference set consisting of 53 nucleosomes into two equal-sized sets, and each of which was referred to as either training set or test set in the subsequent 2-fold prediction. Training set was used to obtain a new reference nucleosome structure, and nucleosome DNA sequences from test set were used for prediction. In each round of prediction, entirely identical nucleosome DNA sequences (100% sequence identity) derived from different nucleosome core particles were excluded from the test set and only the results for 11 unique sequences were reported. Other legends are the same as in Figure S2.

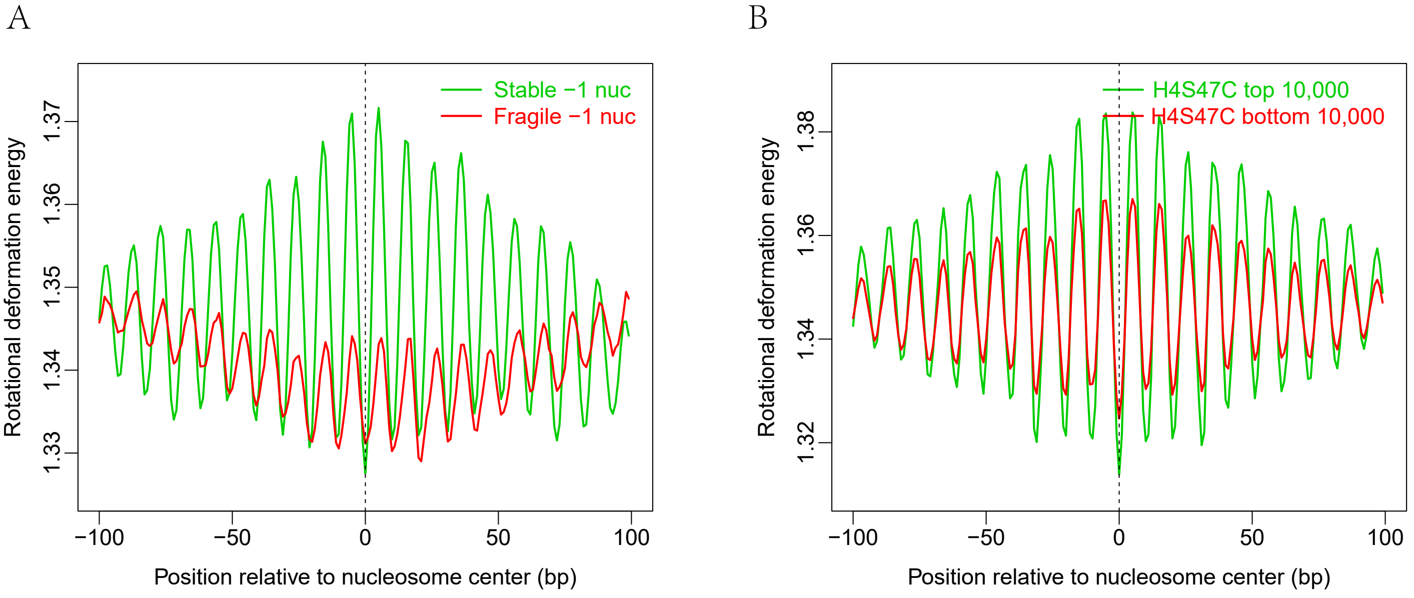

Figure S5. The local minima in rotational deformation energy are predictive of nucleosome-forming preference. (A), rotational deformation energy for fragile -1 nucleosomes and stable -1 nucleosomes (Kubik et al. 2015); (B), rotational deformation energy for low NCP-score (Nucleosome Center Positioning Score) group and high NCP-score group (Brogaard et al. 2012).

Supplementary Methods:

1. Grand canonical model for nucleosome positioning

As described previously (Liu et al. 2016; Morozov et al. 2009), nucleosome positioning is modeled as a 147-bp particle distributed along a genomic sequence of N-bp in length. The probability of a nucleosome to start at genomic position *j* is estimated as

$$P_j = \frac{Z_{j-1}^f e^{-\beta(E_j-\mu)} Z_{j+M}^r}{Z} \tag{1}$$

where μ is chemical potential, E_j is the DNA deformation energy (normalized by sequence length, eg. Energy per base-pair step) of the nucleosome which occupies positions *j* through $j+146$, Z_j^f and Z_j^r are partial partition functions that can be computed in a dynamic programming manner in forward and reverse direction, respectively, and $Z_N^f = Z_1^r = Z$.

The nucleosome occupancy at position *j* is defined as the probability that base-pair *j* is covered by any nucleosome:

$$O_j = \sum_{i=j-146}^{j} P_i \tag{2}$$

In the model, inverse temperature β determines the variance of genome-wide nucleosome occupancy and chemical potential μ is used to simulate nucleosome concentration (and therefore the genome-wide nucleosome occupancy). We re-write $e^{-\beta(E_j-\mu)}$ as $e^{-\beta(E_j-\mu)} = e^{\beta(\mu_1+\overline{E}_j-E_j)} = e^{\beta\mu_1}e^{\beta(\overline{E}_j-E_j)} = \tau e^{\beta(\overline{E}_j-E_j)}$, then the parameter β and τ along with an artificial energy barrier imposed at promoter NDRs are trained to reproduce the regularly spaced nucleosomes around promoter NDRs ($\beta=5$, $\tau=0.1$) with a genome-wide nucleosome coverage of ~70%. The energy barrier is depicted as the following figure. The energy barrier consist of flat energy profile flanked by two half Gaussian shape boundaries (h=0.5, sigma=25). Because there are alternative nucleosomes positioned around the dominant +1/-1 nucleosome centers in different cells, we shifted the borders of the energy barrier 33-bp away from the +1/-1 nucleosome centers, leading to a simulated peak of nucleosome occupancy at experimentally determined +1 nucleosomes (Figure 5B). Note that the height of the energy barrier (h=0.5) is relative to genome-wide average of deformation energies per base-pair-step, and larger than 95% deformation energies to be a "barrier". Non-NDR regions have constant energy (genome-wide average of deformation energies) (Figure 5B: constant E+barrier), or adopt sequence-dependent deformation energies (Figure 5B: sequence E+barrier).

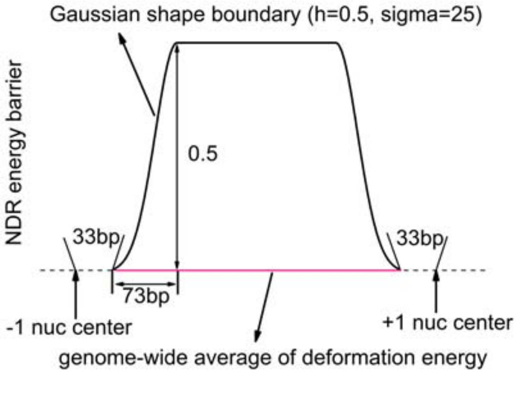

2. Calling unique nucleosomes from the H3Q85C map and mapping MNase-sensitive nucleosomes to the unique map

To test if nucleosome's MNase-sensitivity is caused by the unfavorable deformation energy profile or by AT-richness, we need to compare between MNase-sensitive nucleosomes and MNase-insensitive nucleosomes. We classified nucleosomes into such two groups based on H3Q85C map (Chereji et al. 2018). Specifically, we first called, similarly as in Brogaard et al. 2012, a set of unique nucleosomes from the H3Q85C map. In this step, we first obtained an averaged dyad score map by averaging over three H3Q85C replicates, and then used the averaged dyad scores as input of unique nucleosome calling approach (Xi et al. 2014). There is a large number of zeros in the dyad scores and thus to avoid possible bias in estimating noise to nucleosome uniqueness, we did not consider noise term in unique nucleosome calling, but instead directly used the averaged dyad scores. The unique nucleosome calling approach (Xi et al. 2014) was designed to call the first unique nucleosome on each chromosome as the one centering at a genomic position with the highest dyad score. Then the other unique nucleosomes were iteratively called according to the decreasing order of dyad scores, in which adjacent nucleosomes are allowed to overlap at most 40 bp. We obtained in this way a unique nucleosome map composed of 72,438 nucleosomes (Supplementary Table S10). After the unique nucleosome map is obtained, we mapped the previously identified MNase-sensitive nucleosomes (Chereji et al. 2017) to the unique nucleosome set, in which an overlap of at least 74 bp is considered as a match. In this way we obtained 2,443 MNase-sensitive nucleosomes and 68,641 non-sensitive nucleosomes from the unique nucleosome map, and analyzed their AT-content and sequence-based nucleosome occupancy in Fig 6C-D. Note that to avoid potential bias caused by MNase-sensitive non-histone particles (Chereji et al. 2017), unique nucleosomes which have an overlap of at least 74 bp with MNase-sensitive non-histone particles were excluded from our analysis.

As to the rotational deformation energy analysis in Fig S5A, accurate identification of nucleosome center is of critical importance. To do this, we first called a redundant nucleosome set from the H3Q85C map (Chereji et al. 2018) by using the unique nucleosome calling approach (Xi et al. 2014) with a modification that adjacent nucleosomes are allowed to overlap at most 137 bp. This means the allowed minimal center-center distance between adjacent nucleosomes is 10 bp, suggesting that this nucleosome map is much more redundant than the aforementioned unique map. This redundant map is used to identify the centers of -1 nucleosomes in Fig S5A. In other words, -1 nucleosomes identified in MNase-seq map (Kubik et al. 2015) were mapped to the redundant map, and an overlap of at least 137 bp was considered as a match. The mapped – 1 nucleosomes have both accurate translational positioning and rotational setting, which could increase the reliability of rotational deformation energy analysis.

References:

Brogaard K, Xi L, Wang JP, Widom J (2012) A map of nucleosome positions in yeast at base-pair resolution. Nature 486:496-501.

Chereji RV, Ocampo J, Clark DJ (2017) MNase-sensitive complexes in yeast: nucleosomes and non-histone barriers. Mol Cell 65:565-577.

Chereji RV, Ramachandran S, Bryson TD, Henikoff S (2018) Precise genome-wide mapping of single nucleosomes and linkers in vivo. Genome Biol 19:19.

Kaplan N, Moore IK, Fondufe-Mittendorf Y, Gossett AJ, Tillo D, Field Y, LeProust EM, Hughes TR, Lieb JD, Widom J, Segal E (2009) The DNA-encoded nucleosome organization of a eukaryotic genome. Nature 458:362-366.

Kubik S, Bruzzone MJ, Jacquet P, Falcone JL, Rougemont J, Shore D (2015) Nucleosome stability distinguishes two different promoter types at all protein-coding genes in yeast. Mol Cell 60:422-434.

Liu G, Xing Y, Zhao H, Wang J, Shang Y, Cai L (2016) A deformation energy-based model for predicting nucleosome dyads and occupancy. Sci Rep 6:24133.

Morozov AV, Fortney K, Gaykalova DA, Studitsky VM, Widom J, Siggia ED (2009) Using DNA mechanics to predict in vitro nucleosome positions and formation energies. Nucleic Acids Res 37:4707-4722.

Xi L, Brogaard K, Zhang Q, Lindsay B, Widom J, Wang JP (2014) A locally convoluted cluster model for nucleosome positioning signals in chemical map. J Am Stat Assoc 109: 48-62.
